# Tunable Micro- and Nanomechanical Resonators

**DOI:** 10.3390/s151026478

**Published:** 2015-10-16

**Authors:** Wen-Ming Zhang, Kai-Ming Hu, Zhi-Ke Peng, Guang Meng

**Affiliations:** State Key Laboratory of Mechanical System and Vibration, School of Mechanical Engineering, Shanghai Jiao Tong University, 800 Dongchuan Road, Shanghai 200240, China; E-Mails: hukaiming@sjtu.edu.cn (K.-M.H.); z.peng@sjtu.edu.cn (Z.-K.P.); gmeng@sjtu.edu.cn (G.M.)

**Keywords:** MEMS/NEMS, micromechanical resonator, nanomechanical resonator, frequency tuning, tuning process

## Abstract

Advances in micro- and nanofabrication technologies have enabled the development of novel micro- and nanomechanical resonators which have attracted significant attention due to their fascinating physical properties and growing potential applications. In this review, we have presented a brief overview of the resonance behavior and frequency tuning principles by varying either the mass or the stiffness of resonators. The progress in micro- and nanomechanical resonators using the tuning electrode, tuning fork, and suspended channel structures and made of graphene have been reviewed. We have also highlighted some major influencing factors such as large-amplitude effect, surface effect and fluid effect on the performances of resonators. More specifically, we have addressed the effects of axial stress/strain, residual surface stress and adsorption-induced surface stress on the sensing and detection applications and discussed the current challenges. We have significantly focused on the active and passive frequency tuning methods and techniques for micro- and nanomechanical resonator applications. On one hand, we have comprehensively evaluated the advantages and disadvantages of each strategy, including active methods such as electrothermal, electrostatic, piezoelectrical, dielectric, magnetomotive, photothermal, mode-coupling as well as tension-based tuning mechanisms, and passive techniques such as post-fabrication and post-packaging tuning processes. On the other hand, the tuning capability and challenges to integrate reliable and customizable frequency tuning methods have been addressed. We have additionally concluded with a discussion of important future directions for further tunable micro- and nanomechanical resonators.

## 1. Introduction

With the rapid advance of the micro- and nanotechnologies in micro/nano-electro-mechanical systems (MEMS/NEMS), more and more micro- and nanomechanical resonators have been developed, which are of interest to both the scientific community and engineering fields due to their significant advantages such as small size, compactness, high sensitivity, high resolution, low power consumption and low cost, and high quality factor [1,2,3,4,5]. Due to their small sizes, micro- and nanomechanical resonators can oscillate at very high resonant frequencies, which provides them with a remarkable ability to perform both sensing and detection in advanced technological applications, including ultrasensitive mass and force sensing, ultralow-power radio frequency (RF) signal generation and timing, chemical and biological sensing, cooling, environmental control, and quantum measurement [6,7,8,9,10,11,12]. However, there still exist fundamental and technological challenges to tunable micro- and nanomechanical resonators.

In general, different techniques for designing the micro- and nanomechanical resonators can be categorized into both vibration-based methods and wave propagation-based methods [13]. The fundamental characteristics of mechanical resonators are determined by the resonant frequency and quality factor (energy dissipation). As one of the important attributions to resonating MEMS/NEMS devices, resonant frequency often determines the sensitivity and accuracy of the system. Various micro- and nanomechanical resonator applications, such as high resolution sensors, RF oscillators and filters, can be benefit from the tuning capability of resonant frequency or operation range, which allows fabrication of multi-functional components for multi-band filtering, has low power temperature compensation targeted for timing reference and RF synthesizing applications [14,15,16,17,18,19,20,21,22,23,24,25,26,27,28]. The most desirable function is the tunability of the resonant frequency, which can be used to compensate for the resonant frequency shift in resonators due to the changes in temperature, pressure, or atmosphere composition [14]. An interesting application frequency tuning is the ability to controllably couple the out-of-plane and in-plane vibration modes as the frequencies of the two modes are tuned closer to each other [15]. It can be used to optimize frequency and nonlinearity tuning and to increase the pull-in threshold for specific applications of small and sensitive devices as linear sensors. Frequency tuning on short timescales [16] can be necessary for mechanical signal processing which requires signal tracking, frequency hopping, *etc.* Moreover, frequency tuning can be applied when the structure dimensions of the resonators changes due to the fabrication process [16], can be useful in controlling frequency instability and deterministic switching between bistable states [17], and can realize controllable sensitivity [18]. Therefore, the ability to tune the resonant frequency of a micro- and nanomechanical resonator is crucial for potential applications. The fundamental understanding of the frequency tuning mechanisms becomes important for the future design and optimization of micro- and nanomechanical resonators in the very-high frequency (VHF), ultra-high frequency (UHF) ranges.

The resonant frequency of micro- and nanomechanical resonators depend upon many factors, including geometry, structural material properties, stress, external loading, and surface topography. Many methods have been proposed to guarantee frequency tuning throughout the lifetime of micro- and nanomechannical resonators, and overcomes the relative drawbacks arouse from the nonlinear effect, environmental effect and fabrication related effects such as processing temperatures, fabrication tolerances, structural non-idealities and asymmetries, residual stress as well as design errors and defects which can cause resonant frequency shift [19,20,21,22]. Therefore, it is important to develop tuning methods which depend on the change of the stiffness or mass of the mechanical resonators. Inducing stresses in the resonator can change its effective stiffness and the resonant frequency [14]. Since micro- and nanomechanical resonators are characterized by a large surface-to-volume ratio, it is demonstrated that the surface phenomena plays a significant role on not only the resonance behavior but also the sensing or actuating performance of the devices [23,24]. In addition, micro- and nanomechanical resonators have widely implemented in various fluidic environments, as a result, the viscous fluids lead to the shift of resonant frequency in the resonators and the fluid-structure interaction causes the challenge to perform measurement in viscous fluids [25,26].

Various geometry structures like cantilever and bridge beams, and plates are the most typical micro- and nanomechanical resonators. Recent advance of fabrication technologies leads to the increasing complexity of resonating devices which generate challenges to potential resonator applications [27]. Micro- and nanomechanical resonators using the structures and materials such as tuning electrode [28], tuning fork [29], suspended channel structure [30], carbon nanotubes [31], nanowires [32], graphene sheets [33] and bulk micromachined structures [34], as well as the smallest man-made self-assembled molecular structure [35] provide the promise of new applications and allow us to explore fundamental properties at the micro- and nanoscales. It is of great interest to tune and control their resonant frequencies reversibly.

Frequency tuning methods can be usually divided into two major categories: active and passive methods [36]. Many researchers have extensively developed various methodologies to address the changes by correcting the resonant frequency using tuning procedures. Active tuning is defined as a tuning mechanism that is continuously applied even if the resonant frequency closely matches the excitation vibration frequency [37]. Real time tuning makes these methods very attractive and some active methods, including electrothermal [14,19], electrostatic [38,39,40], magnetomotive [41], piezoelectrical [42,43], dielectric [44], photothermal [45], and modal coupling [46,47], as well as the tension-induced tuning mechanisms, have been developed and reported. In contrast, passive tuning method often operates periodically and only consumes power during the tuning operation [37]. The manufacturing variations due to fabrication processes may cause discrepancies in designed specifications, which should be needed to compensate using the post-fabrication tuning processes [48]. However, these methods are unable to implement real time frequency tuning for mechanical resonators throughout their lift times. Furthermore, zero on-chip energy consumption makes passive methods favorable for low power applications [36].

During the past several years, some extensive and critical reviews on micro- and nanomechanical resonators and comprehensive analyses of their wide rage for MEMS/NEMS applications have reported [49,50,51], such as the recent reviews on carbon nanotube and graphene-based nanomechanical resonators [2,13,52,53,54], microcantilever-based resonator applications and sensing principles [55], nanomechanical resonators and their applications in biological/chemical detection [56] and visualization of material structure [57], nonlinear dynamics and its applications in micro-and nanomechanical resonators [58], MEMS-based oscillators for frequency reference applications [59], micromechanical resonators applied in vibration energy harvesters [60], cantilever-like micromechanical resonators for recent sensor applications [61], nanomechanical resonators for all-optical mass sensing [11], dissipation in nanomechanical resonator structures [62], some fundamental and nonfundamental noise processes limited the performance of nanomechanical resonators [63], and an outlook of how state-of-the art mechanical resonators can be improved to study quantum mechanics [64]. The current review focuses on the methodologies of frequency tuning for micro- and nanomechanical resonators. The purpose of this review is to present not only the current state-of-the-art in the development of frequency tuning methods for micro- and nanomechanical resonator applications, but also the resonant frequency shift due to the major influencing factors that have enabled fundamental insights into the frequency tuning principles and mechanisms as well as the some novel tuning structures and tunable resonators.

This review is organized as follows: In Section 2, we present the theoretical description of the resonance behavior in the flexural and torsional modes of vibration of the beam-based mechanical resonators. Section 3 provides the brief overview of the frequency tuning principle for the resonators, in which the flexural and torsional operation modes of motion are described via a basic mechanical model. Except for the typical beam/plate-based micro- and nanomechanical resonators, an overview of some novel tuning structures such as tuning electrodes, tuning fork and suspended channel, and newly tunable resonators made from graphene are reviewed and discussed in Section 4. Performance issues such as sensitivity, stability and resolution are also addressed. Section 5 focuses on the major influencing factors, such as large-amplitude effect, surface stress effect and fluid effect, which affect the resonant frequency shift in resonators, and briefly review the efforts implemented to predict, control and apply the resonant frequency shift for overcoming challenges and potential applications. The active and passive tuning mechanisms, methods and techniques, and extending applications are reviewed in detail and discussed in Section 6 and Section 7. A perspective on future challenges and conclusion remarks are concluded in Section 8.

## 2. General Resonance Behavior

The resonant frequency of any mechanical resonator is determined primarily by the geometrical dimensions, structural material properties, stress, and surface topography. To clearly understand the general resonance behavior, we briefly review the flexural vibration and torsional vibration modes in beam-based resonators in this section.

### 2.1. Flexural Vibration Modes

#### 2.1.1. Single Layer Beam Model

**Figure 1:**
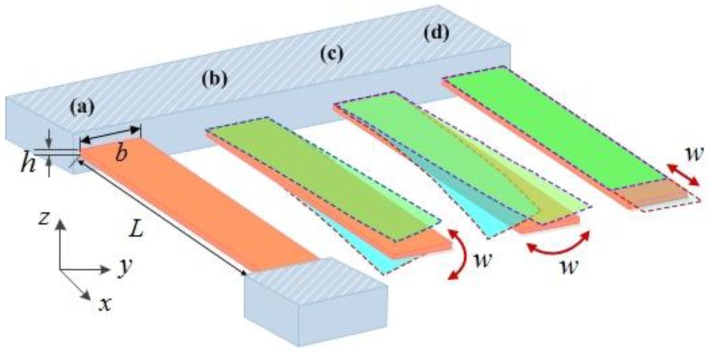
Schematic diagram of the beam-based resonators. Doubly clamped beam (**a**) and cantilever beam with the flexural (out-of-plane) mode (**b**), the lateral (in-plane) bending mode (**c**) and the elongation (in-plane) mode (**d**).

For small amplitudes, the mechanical resonances of beam structures consisting of uniform material can be described analytically by the (Euler–Bernoulli theory [65]. A schematic depiction of the typical cantilever and clamped-clamped beam resonators is shown in Figure 1. For flexural modes of vibration, the governing equation for the elastic deformation of the beam is given by:

(1) $$EI\frac{\partial^{4}w(x,t)}{\partial x^{4}}+\rho A\frac{\partial^{2}w(x,t)}{\partial t^{2}}-N\frac{\partial^{2}w(x,t)}{\partial x^{2}}=f(x,t)$$

where w(x,t) is the time dependent transverse displacement of the beam in the z direction, *E* is Young’s modulus, *ρ* is the density, *I* and *A* are the moment of inertia and cross-sectional area of the beam, respectively, *N* denotes the axial (tensile) force, f(x,t) indicates the external driving force per unit length, *x* is the spatial coordinate along the length of the beam, and *t* is time.

The corresponding boundary conditions for the cantilever and clamped-clamped beams are:

(2) $${\left[w(x,t)=\frac{\partial w(x,t)}{\partial x}\right]}_{x=0}={\left[\frac{\partial^{2}w(x,t)}{\partial x^{2}}=\frac{\partial^{3}w(x,t)}{\partial x^{3}}\right]}_{x=L}=0$$

and:

(3) $${\left[w(x,t)=\frac{\partial w(x,t)}{\partial x}\right]}_{x=0}={\left[w(x,t)=\frac{\partial w(x,t)}{\partial x}\right]}_{x=L}=0$$

To calculate the resonant frequency of the beams, we can assume a harmonic transverse vibration given by w(x,t)=W(x)exp(−iωt) in the absence of axial force N=0 and external force f(x,t)=0, the general solution for the beam displacement can be given by:

(4) $$w(x,t)=C_{1}\cos\beta x+C_{2}\sin\beta x+C_{3}\cosh\beta x+C_{4}\sinh\beta x$$

where $C_{1}\sim C_{4}$ are the various constants, $\beta^{4}=\rho A\omega^{2}/(EI)$, and the parameter *β* takes discrete values satisfying the relations, $\cos\beta_{n}L\cosh\beta_{n}L+1=0$ and $\cos\beta_{n}L\cosh\beta_{n}L-1=0$ in which *n* is the mode order, for the cantilever and doubly clamped beams, respectively.

Then, the well-known result for the resonant frequencies of the corresponding modes can be given by:

(5) $$\omega_{n}=2\pi f_{n}=\beta_{n}^{2}\sqrt{\frac{EI}{\rho A}}=\frac{\lambda_{n}^{2}}{L^{2}}\sqrt{\frac{EI}{\rho A}}$$

where $\lambda_{n}=\beta_{n}L=1.875,4.694,7.855,10.996,\ldots$ and $\lambda_{n}=\beta_{n}L=0,4.730,7.853,10.996,14.137,\ldots (n=0,1,2,3\cdots )$ for the cantilever and doubly clamped beams, respectively. In addition, calibration of non-rectangular cantilever beam or beam with uniform arbitrary cross section is of increasing importance [66,67]. Doubly clamped beams have higher resonant frequencies than cantilever beams of the same dimensions.

**Figure 2:**
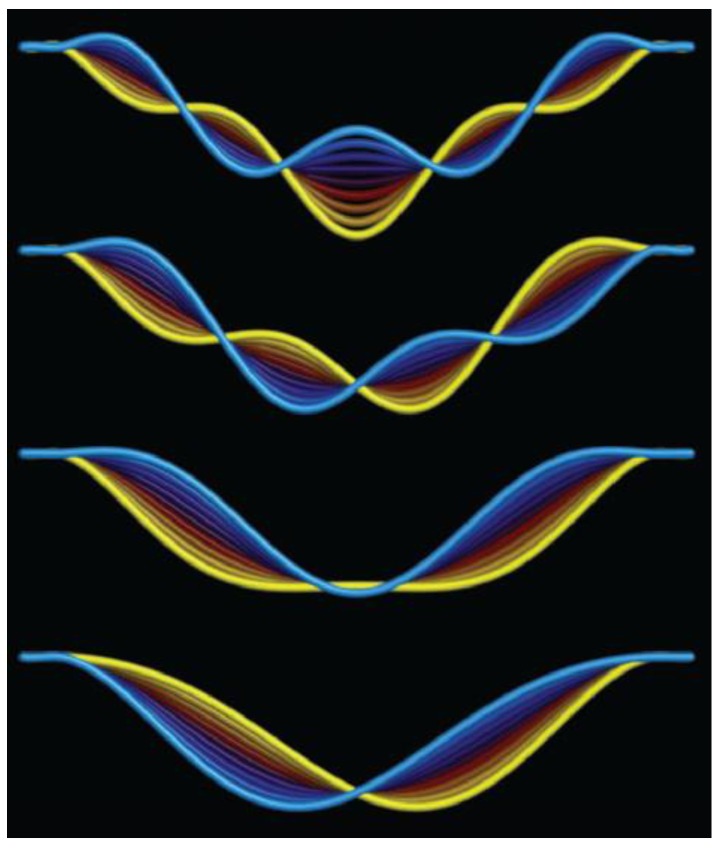
First four resonant modes of a carbon nanotube. Reused with permission from [73], Copyright 2004 Nature Publishing Group.

Carbon nanotubes (CNTs) have a high Young’s modulus and excellent stiffness combined with low mass [54,68]. The reported resonance frequencies of CNTs vary from several MHz to a few hundred MHz [54]. The implication is that a beam can vibrate in certain vibrational modes with the distinct spatial shapes. The first four vibrational mode-shapes of a carbon nanotube are shown in Figure 2. It can be seen that certain areas of the nanotube have large vibrational amplitude while other areas are fluctuating with low amplitude. The number of nodal points increases with increasing mode number. Sazonova *et al*. [31] have detected such vibrations and demonstrated their tunability of both single and multiple resonances over a range of frequencies from 5 to 150 MHz in carbon nanotube. It was demonstrated that single-walled carbon nanotube (SWCNT) nanomechanical resonators can serve as mass sensing that are capable of detecting individual atoms or molecules [69,70,71]. Liu *et al*. [72] also investigated the first five out-of-plane resonant modes of a single-crystal paddle resonator. The measured resonant frequency of the torsional mode is about 43.762 kHz, while these of the flexural modes (1–1 and 2–0) are 470.458 kHz and 518.463 kHz, respectively.

When the width of the beam structure is considerably larger than its thickness, *i.e*., b>5h, the effective Young’s modulus $\overset{⌢}{E}$ instead of E should be used to calculate the resonant frequency for such thin beams and written as:

(6) $$\overset{⌢}{E}=E/(1-\nu^{2})$$

where *v* is the Poisson’s ratio to account for the suppression of the in-plane dilatation accompanying axial strain.

#### 2.1.2. Multilayer Beam Model

Multilayer beam-based micro- and nanomechanical resonators often use sensitive coating or piezoelectric layers for mass and gas detections [74]. Most of them have a variable cross-section that complicates the prediction the resonant frequency using conventional beam models [75,76,77,78,79]. The Euler-Bernoulli differential equation solutions provide the resonant frequency of a composite beam with uniform cross-sectional by replacing the bending stiffness EI and density ρ with the composite bending stiffness $\bar{\mathrm{EI}}$ and composite density $\bar{\rho }$ [75,76]. The resonant frequency of a composite beam with *N* layers can be written as:

(7) $$\omega_{n}=2\pi f_{n}=\beta_{n}^{2}\sqrt{\frac{\bar{\mathrm{EI}}}{\bar{\rho }A}}=\frac{\lambda_{n}^{2}}{L^{2}}\sqrt{\frac{\bar{\mathrm{EI}}}{\bar{\rho }A}}$$

where:

(8) $$\bar{\rho }=\sum_{i=1}^{N}\rho_{i}t_{i}/\sum_{i=1}^{N}t_{i}$$

where $t_{i}$ is the thickness of the individual layers, $I_{i}$ is the individual moment of inertia for each layer and can be computed by:

(9) $$I_{i}=\frac{bt_{i}^{3}}{12}+A_{i}d_{i}^{2}$$

where *b* is the width of the top layer, $A_{i}$ is the cross-sectional area of the individual layer, $d_{i}$ is the distance between the centroidal axis of the composite beam and the neutral axis of each individual layer. The width of each normalized layer $b_{i}$ can be calculated using the transformed-section method to be $b_{i}=E_{i}b/E_{N}$, where $E_{i}$ is the Young’s modulus of each layer, and $E_{N}$ is the Young’s modulus of the top layer. Melamud *et al*. [77] presented the nominal mechanical resonant frequency of the composite beam resonator composed of three structural materials. The combination of the resonant frequency can be given by $f_{n,\text{norm}}^{2}=\sum_{i}m_{i}f_{i}^{2}/m_{r}$ i=1,2,3,⋯), where $f_{i}$ and $m_{i}$ are the resonant frequency and mass of the composite structural materials, respectively.

### 2.2. Torsional Vibration Modes

To clearly describe the torsional mode in micro- and nanomechanical resonators, a general theoretical model is introduced for the torsional vibration of a cantilever beam. The governing equation of a cantilever beam undergoing torsional deformation can be given by [80,81]:

(10) $$GK\frac{\partial^{2}\Phi (x,t)}{\partial x^{2}}-\rho_{C}I_{p}\frac{\partial^{2}\Phi (x,t)}{\partial t^{2}}-=M(x,t)$$

where Φ(x,t) is the deflection angle about the major axis of the cantilever, *G* is the shear modulus of the cantilever, *K* is a geometric function of the cross section of the beam, $\rho_{C}$ is the density, $I_{p}$ is the polar moment of initial, and M(x,t) indicates the applied torque per unit length along the beam. The corresponding boundary conditions are [81]:

(11) $$\Phi (0,t)={\frac{\partial \Phi (x,t)}{\partial x}|}_{x=L}=0$$

Following a similar analysis to that implemented for the flexural modes [81], the Fourier transform of Equation (10) can be written as:

(12) $$GK\frac{d^{2}\overset{⌢}{\Phi }(x|\omega )}{dx^{2}}+\rho_{C}\omega^{2}I_{p}\overset{⌢}{\Phi }(x|\omega )=\overset{⌢}{M}(x|\omega )$$

where the Fourier transform of any function x(t) is expressed as $\overset{⌢}{X}(\omega )=\int_{-\infty }^{\infty }x(t)e^{i\omega t}dt$. Then the resonant frequency in vacuum can be given by [80,81]:

(13) $$\begin{array}{cc} \omega_{n}=2\pi f_{n}=\frac{D_{n}}{L}\sqrt{\frac{GK}{\rho_{c}I_{p}}}, & D_{n}= \end{array}\frac{\pi }{2}(2n-1)$$

where n=1,2,3,… is the mode order. For a thin rectangular beam, $K=bh^{3}/3$ and $I_{p}=b^{3}h/12$. The typical torsional Cleveland method and torsional Sader method are briefly reviewed and discussed in [82]. Hall *et al*. [83] provided a brief review of CNT torsional resonating devices which hold particular potential for biological and chemical mass sensing [69,71].

### 2.3. Fundamental Resonant Frequency

Different structure dimensions play very important role in the resonant frequency [84]. The fundamental resonant frequency of a structure can be determined by both its dimensions and mechanical properties of the material. The analytical formulas for the fundamental resonant frequencies of cantilever beams $f_{0}^{CB}$, clamped-clamped beams $f_{0}^{CC}$ and circular disks $f_{0}^{CD}$ are expressed as [84,85]:

(14) $$f_{0}^{CB}=0.162(h/L^{2})\sqrt{E/\rho }$$

(15) $$f_{0}^{CC}=1.03(h/L^{2})\sqrt{E/\rho }$$

(16) $$f_{0}^{CD}=1.65(h/{D_{d}}^{2})\sqrt{E/\rho }$$

where *E* and *ρ* are the Young’s modulus and the density, respectively, *h* is the thickness of the structure, *L* is the length of the beam, and $D_{d}$ is the diameter of the disk.

From the comparison of Equations (14)–(16), it can be found that for similar dimensions, circular disks present a resonant frequency approximately ten times higher than cantilever beams and about 1.6 times higher than clamped-clamped beams. In addition, the effects of internal and residual stresses due to the resonator’s material, its design and the fabrication process on the resonant frequency should be taken into account [84,85].

## 3. Principle of Frequency Tuning

Resonant devices, such as vibrating beams, plates and diaphragms are widely used for micro- and nanosensor and actuator applications, in which the precise tuning of the resonant frequency is very important. Previous frequency tuning methods relied on changing either the stiffness, or the mass of the resonators. To briefly introduce and discuss the principle of frequency tuning for resonators, flexural (translational) and torsional modes of motion of the beam-based resonators are described in this section.

### 3.1. Basic Mechanical Model

**Figure 3:**
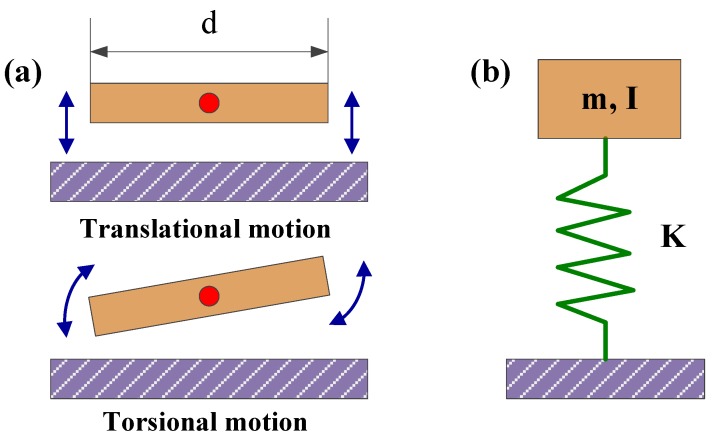
Principle diagram and two mods of motion of a paddle resonator. (**a**) Schematic diagrams of translational and torsional modes of motion; (**b**) Equivalent lumped-parameter model.

Lumped-parameter modeling enables analyzing this structure as a single degree-of-freedom, Evoy *et al*. [86] presented meaningful models for the flexural (translational) and torsional modes of motion of a typical paddle resonator, as shown in Figure 3.

The resonant frequency of a damped oscillator can be derived from the lumped-parameter model (Figure 3b). For the translational mode, the equation of motion can be given by:

(17) $$m\ddot{x}+C\dot{x}+Kx=F(t)$$

where *m*, *C* and *K* are the mass, the damping, and the spring constant of the system, respectively, and *F* is the external force. Using the lumped parameter model, the fundamental resonant frequency of the beam structure can be expressed as:

(18) $$f_{0}=\frac{1}{2\pi }\sqrt{\frac{k_{\text{eff}}}{m_{\text{eff}}}}$$

where $k_{\text{eff}}$ and $m_{\text{eff}}$ are location-dependent, and the effective stiffness and mass of the resonator, respectively. The mass and the spring constant are the effective parameters to control the resonant frequency. For the micromechanical resonators, which are fabricated using batch micromachining processes that entail successive steps of film deposition, lithography and etching, their frequencies are strongly dependent on the absolute and matching tolerances of these steps [38]. These finite tolerances lead to variations in dimensions and stress, resulting in $k_{\text{eff}}$ and $m_{\text{eff}}$ deviations that then offset the final fabricated resonant frequency from the desired design frequency. In the interest of maintaining a simple formulation, the frequency of the fabricated device can be given by:

(19) $$f_{0}=\frac{1}{2\pi }\sqrt{\frac{k_{\text{eff}}+\Delta k}{m_{\text{eff}}+\Delta m}}$$

where Δ*k* and Δ*m* are stiffness and mass offset coefficient, respectively, generated by finite fabrication tolerances.

For the torsional mode, the equation of motion can be given by [86]:

(20) $$I\ddot{\theta }+(I/\omega_{0}Q)\dot{\theta }+K\theta =\tau (\theta ,t)$$

where *I* is the inertial of the paddle, and *K* is the torsional spring constant and $K=2GI_{p}/L$, where *G* denotes the modulus of elasticity in shear, $I_{p}$ is the polar moment of inertia of the area, and *L* is the equivalent length of the bar. The resonant frequency of the resonator can be written as:

(21) $$f_{0}=\frac{1}{2\pi }\sqrt{\frac{K}{I}}$$

The two resonant frequencies are preliminary attributed to the excitation of translational and torsional modes of motion, and $f_{\text{0}}^{\text{translational}}\propto d^{-0.5}$ and $f_{\text{0}}^{\text{torsional}}\propto d^{-1.5}$ in theory. A fit of measurement data a $f_{\text{0}}^{\text{measurement}}=Kd^{\vartheta }$ power law reveals experimental power coefficients of ϑ=−0.5±0.1 and ϑ=−1.6±0.15 for translational and torsional modes [86], respectively. The resonant frequency of the resonator is affected by the external force and bending moment on the resonance structure, and the force and the moment can be determined by the changing frequency. Therefore, the resonant frequency of the resonator can be tuned by controlling the force or the moment.

### 3.2. Mass Tuning

**Figure 4:**
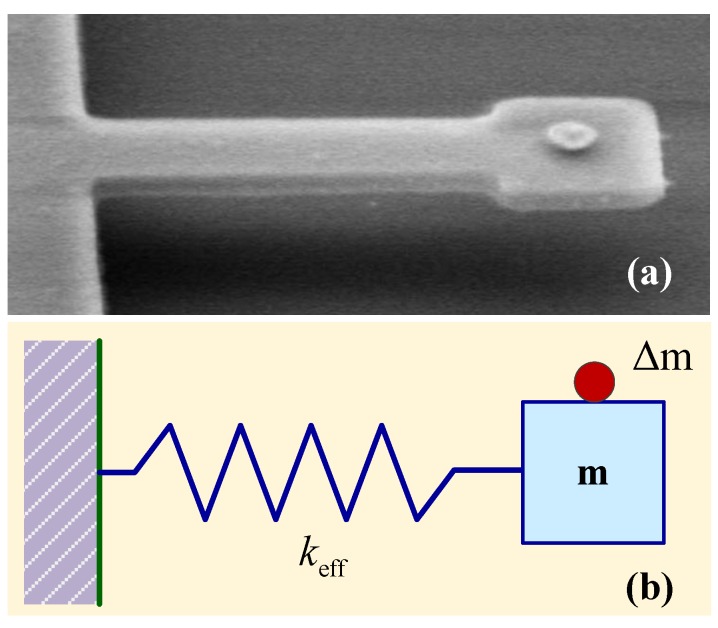
(**a**) SEM of a clamped-free beam resonator with added mass at the tip. Reused with permission from [87]; (**b**) Schematic of the undamped spring-mass system with added mass effect.

Micro- and nanomechanical resonators have frequencies of vibration that are sensitive to small amounts of added mass. As one of the typical micro- and nanomechanical resonators, cantilever structure have been proposed for highly sensitive detection of organic and biological molecules [23,87].The basic principle is the measurement of the resonance frequency shift due to the added mass on the cantilever surface [88]. The change in resonant frequency of the resonator can be modeled by an undamped spring-mass model, as shown in Figure 4. Any additional mass Δm can result in resonant frequency reduction, and the resonant frequency can be expressed as $f_{CB}=0.162h/L^{2}\sqrt{E/(\rho (1+4\gamma_{a}))}$ [87], where $\gamma_{a}$ is the ratio of the added mass to the mass of the beam. The frequency change Δf can be given by:

(22) $$\Delta f=\frac{f_{\text{add}}-f_{0}}{f_{0}}=\sqrt{\frac{m}{m+\Delta m}}-1$$

where $f_{0}$ and $f_{\text{add}}$ are the resonant frequency before and after adding the mass, such as PLD process [89], adsorption [23]. The negative resonant frequency shift due to the NC-DNA is related to the mass added by the adsorption near the cantilever tip [23]. The cantilevers with the gold and Au areas on the free end exhibited the resonant frequency of about 310 kHz and 650 kHz, respectively. The measured resonant frequency shifts were 125 Hz and 1.10 kHz corresponding to the added masses of 6.3 and 213.1 ag [87], respectively. The added mass of the adsorbed bacteria leads to a negative resonant frequency shift [90]. Therefore, the structure material and weight of the added mass play an important role on the resonant frequency shift of the resonators.

In addition, Yi *et al*. [91] have systematically investigated the resonant frequency shift of the cantilever due to the added mass effect with various distribution conditions, as listed in Table 1. The resonant frequency shift per unit added mass depends on the state of the mass distributed on the beam. Δf/Δm distributed over the entire cantilever surface is reduced to 0.236 times that obtained with Δ*m* being a point mass or a narrow strip at the tip. The same scaling relationship can be used to a strip mass added at the tip as well as uniformly distributed mass on the cantilever surface.

**Table 1:** Effect of added mass on the resonant frequency shift of the cantilever beam reported by Yi *et al*. [91]. Reused with permission from [91].

Description	Added Point Mass at the Tip	Added Mass Distributed on a Narrow Strip	Added Mass Distributed over the Entire Beam	Size Reduction
Schematic	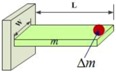	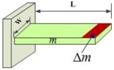	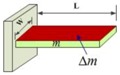	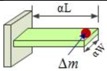
Resonant frequency shift	$\frac{\Delta f}{\Delta m}≅\frac{\nu_{n}^{2}}{4\pi }\frac{1}{L^{3}W}\left(\frac{1}{0.236\sqrt{12}\rho }\sqrt{\frac{E}{\rho }}\right)$		$\frac{\Delta f}{\Delta m}≅\frac{\nu_{n}^{2}}{4\pi }\frac{1}{L^{3}W}\left(\frac{1}{\sqrt{12}\rho }\sqrt{\frac{E}{\rho }}\right)$	$\frac{\Delta f}{\Delta m}\left(\alpha L,\alpha W\right)=\alpha^{-4}\frac{\Delta f}{\Delta m}\left(L,W\right)$

#### 3.2.1. Fixed Mass: Deposition/Adsorption

**Figure 5:**
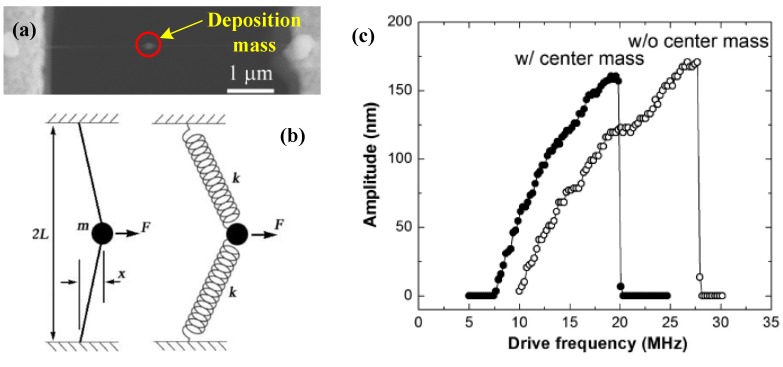
Dynamic response of the CNT nonlinear nanomechanical resonator with the added mass effect reported by Cho *et al*. [92]. (**a**) SEM image of the Pt deposit in the middle of a suspended CNT resonator; (**b**) Schematic diagram of a simple doubly clamped mechanical beam model with the intrinsic geometric nonlinearity; (**c**) The dynamic response of the resonator without and with depositing a center mass with electron-beam-induced deposition. Reused with permission from [92], Copyright 2010, American Chemical Society.

To evaluate the added mass effect on the dynamic behavior of the nanomechanical resonators, Cho *et al*. [92] simply introduced the intrinsic nonlinearity into the nanomechanical resonators via a geometric design, as shown in Figure 5. A small amount of platinum was deposited on the middle of the CNT with electron-beam-induced deposition (Figure 5a). Considering the geometric nonlinearity induced by axial tension and added mass effect, the vibration of the beam can be given by [92]:

(23) $$\left[\rho A+m_{c}\delta (x-L)\right]\frac{\partial^{2}w(x,t)}{\partial t^{2}}+EI\frac{\partial^{4}w(x,t)}{\partial x^{4}}+\frac{m\omega_{0}}{Q}\frac{\partial w(x,t)}{\partial t}-N\frac{\partial^{2}w(x,t)}{\partial x^{2}}=F_{m}\cos\omega t\delta (x-L)$$

where $m_{c}$ is the added mass attached to its middle position, $F_{m}$ is the transverse point force applied to the middle of the wire.

The ratio of the drop frequency to the resonant frequency can be expressed as:

(24) $$\Delta f_{ratio}=\frac{f_{\text{drop}}}{f_{0}}={\left(\frac{1+\sqrt{1+(1+M)\Gamma }}{1+M}\right)}^{1/2}$$

where *M* is the ratio of the added mass to the overall mass of the beam and $M=m_{c}/m_{0}$, $\Gamma =\gamma_{d}{\left(F_{m}Q/E\right)}^{2}{\left(2L/D\right)}^{6}/D^{4}$, in which *D* is the radius of the wire and $\gamma_{d}=0.0303$ [92]. The frequency shift of the nonlinear resonator strongly depends on the added mass effect and the inherent geometric nonlinearity of the beam. The added mass leads to the resonant frequency shift about 2.0 MHz, as illustrated in Figure 5. In addition, the magnitude of the shift in the drop frequency increases with the increase in added mass.

**Figure 6:**
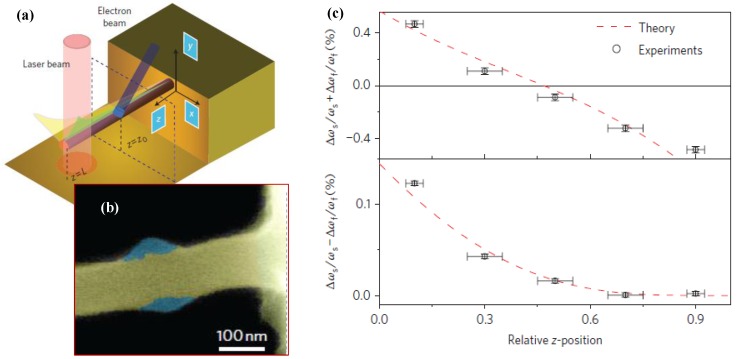
Effect of mass deposition position on the frequency shift in a nanowire resonator reported by Gil-Santos *et al*. [93]. (**a**) Schematic of electron-beam-induced deposition of carbon on nanowires; (**b**) SEM image of the nanowire after electron-beam-induced carbon deposition near the clamped end; (**c**) The sum and difference of the relative frequency shift of a nanowire resonator as a function of the position at which a mass deposited on the nanowire. Reused with permission from [93], Copyright 2010 Nature Publishing Group.

Gil-Santos *et al*. [93] investigated the effect of a molecular adsorbate on the resonant frequency shift in a nanowire resonator, as illustrated in Figure 6. The electron-beam causes slow carbon deposition near the clamed end of a 100-nm-thick nanowire (Figure 6b). The deposition not only leads to the shift of the resonant frequency, but also causes the planes of vibration to rotate. The sum of the relative shift of the frequency and the difference in the relative shift of the frequencies can be written as [93]:

(25) $$\frac{\Delta \omega_{s}}{\omega_{s}}+\frac{\Delta \omega_{f}}{\omega_{f}}≅\frac{V_{D}}{V_{NW}}\left[-\Psi {\left(z_{0}\right)}^{2}\frac{\rho_{D}}{\rho_{NW}}+\Phi {\left(z_{0}\right)}^{2}\frac{E_{D}}{E_{NW}}\right]$$

(26) $$\frac{\Delta \omega_{f}}{\omega_{f}}-\frac{\Delta \omega_{s}}{\omega_{s}}≅\frac{V_{D}}{V_{NW}}\frac{E_{D}}{E_{NW}}\Phi {\left(z_{0}\right)}^{2}\cos(2\beta_{NW})$$

where Ψ and Φ are the non-dimensional eigenmode amplitude and curvature, $E_{D}$, $\rho_{D}$, $V_{D}$ and $E_{NW}$, $\rho_{NW}$, $V_{NW}$ are the Young’s modulus, mass density, and volume of the deposited material and the nanowire, respectively. The added mass dominates as the adsorption approaches the free end. Figure 6c shows the sum and difference of the relative resonant frequency shifts as a function of the longitudinal deposition position. Adsorbate position along the resonator is also known to affect the measurements [23,94,95]. The mass and mechanical properties of the adsorbate can be determined by measuring the sum and difference of the relative frequency shifts [93]. The resonant frequency shift of the resonator depends on not only the adsorbed mass but also the intermolecular interactions [96].

#### 3.2.2. Moveable Mass: Migration

**Figure 7:**
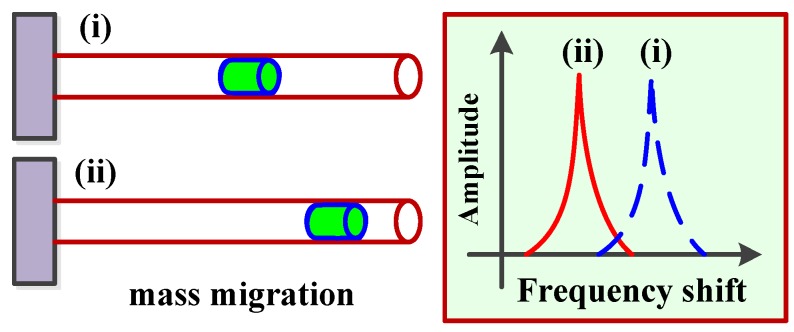
Resonant frequency shift due to mass migration.

Although nanomechanical resonators for mass sensor applications depend on the resonant frequency shifts due to the direct mass adsorption to the resonator, the effective mass of the resonator can be control to tune its resonant frequency, as illustrated in Figure 7.

**Figure 8:**
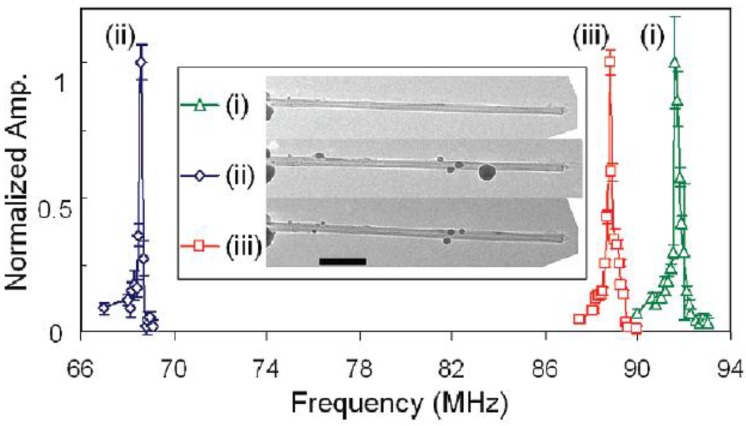
Resonant frequency shifts in MWNT nanomechanical resonators due to mass migration. Reused with permission from [97], Copyright 2009, American Chemical Society.

Kim *et al*. [97] demonstrated reversible frequency tuning of multi-walled carbon nanotube (MWNT) resonator by mass migration method. The resonant frequency of the MWNT is sensitive to the mass distribution of the resonator, as shown in Figure 8. The images in Figure 8 correspond respectively to the unloaded MWNT: (i), after the initial mass loading (ii), and after cleaning and mass reloading (iii). Mass redistribution along the resonator provides reversible tuning with frequency shifts larger than 20% from the initial migration process (ii). The interesting result is the controllability and repeatability of the mass loading process using the current-driven mass migration onto the MWNT. In addition, Kim *et al*. [97] estimated the resonant frequency shift due to the mass adsorption on the MWNT resonator using the Rayleigh-Ritz method. When the masses $m_{i}$ are adsorbed at locations $x_{i}$, the resonant frequency can be expressed as [97]:

(27) $$f_{0}=0.56\sqrt{\frac{EI/L^{3}}{m_{0}+\sum_{i}w(x_{i})m_{i}}}$$

where w(x) is the weighting function and denotes the degree of effectiveness of mass on the resonant frequency. In the case of knowledge of the position $z_{\Delta m}$ and mass Δm of the attached particle (atom, cell or molecule), the resonant frequency can be approximately given by [98]:

(28) $$f_{n}=f_{0}{\left(1+\frac{\Delta m}{m_{0}}U_{n}^{2}(z_{\Delta m})\right)}^{-1/2}$$

where $U_{n}$ is the mode shape, $z_{\Delta m}$ is the position where the mass $m_{0}$ loaded by a point mass Δm.

In addition, the shift in resonant frequency is associated with the location of added mass. Kang *et al*. [99] investigate the CNT-resonator tuned by the effective mass changes via classical molecular dynamics (MD) simulations. The resonant frequencies can be tuned by the position of the encapsulated nanoparticle. The possible resonant frequency-shift-ranges reach 18%–85% via the nanoparticle-position-change. The resonant frequency ratio can be expressed as $f_{n}/f_{0}={\left(1+\Delta m\alpha_{k}{\left(X/L\right)}^{\beta_{k}}\right)}^{-1/2}$, where $\alpha_{k}$ is determined by Δm whereas $\beta_{k}$ relates to the mechanical properties of CNT. The compassion of the experimental results [97], MD simulations [99] and the continuum theory shows that the effective mass change of the cantilever due to the position of the localized mass causes the resonant frequency shift. The effect of linear-mass-density of the encapsulated nanoclusters leads to the range of resonant frequency shift about 22%–45% for different added masses [100]. These good works provide more understanding of the CNT-resonators tuned by the mass migration. It notes that the dephasing of nanomechanical resonators due to the random mass loading of small particles [101] needs to be well understood and qualified for tunable resonator applications.

### 3.3. Stiffness Tuning

**Figure 9:**
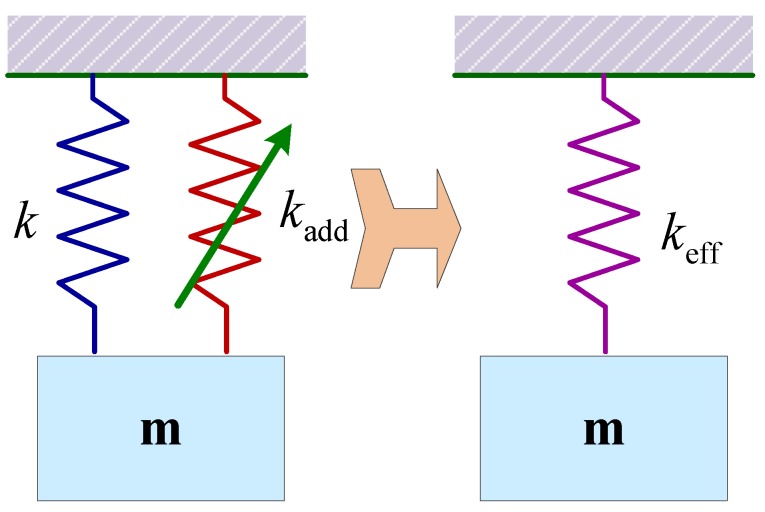
Model of the resonator with softened or hardened spring stiffness.

Spring stiffness tuning, whether hardening or softening, is another common principle to tune the resonant frequency of the resonators [60]. The spring stiffness of the resonator depends on its materials and dimensions. The effective spring stiffness $k_{\text{eff}}$ of such a resonant device can be written as:

(29) $$k_{\text{eff}}=k+k_{\text{add}}$$

where k denotes the mechanical spring constant and $k_{\text{add}}$ is an additional positive or negative spring stiffness due to external loading (thermal, electrostatical, piezoelectrical, magnetical, *etc.*), as illustrated in Figure 9. Then the tuning frequency becomes:

(30) $$f_{tune}=\frac{1}{2\pi }\sqrt{\frac{k+k_{\text{add}}}{m}}$$

The relative tuning methods are described and compared in Section 6 and Section 7. The variable spring stiffness devices are often operated continuously. To clearly understand this principle, one significant work is taken for example, Chen and Hone [53] theoretically analyzed the gate voltage based frequency tuning for graphene mechanical resonators and accurately described the three contributions, including built-in strain, the additional strain upon deformation, and the electrostatic actuation. The effective spring stiffness can be simplified as [53]:

(31) $$k_{\text{eff}}=\frac{16E^{2D}W_{g}\varepsilon_{0}}{3L_{g}}+\frac{256E^{2D}W_{g}}{3L_{g}^{3}}z_{e}^{2}-\frac{1}{2}C^{\prime \prime }V_{g}^{2}$$

where $E^{2D}$ is the 2D Young’s modulus, $W_{g}$ is the width of graphene strip, $\varepsilon_{0}$ is the built-in strain, $L_{g}$ is the length of graphene strip, $z_{e}$ is the maximum static deflection of graphene, $C^{\prime \prime }$ is the second spatial derivative of the capacitance between the graphene and gate, $V_{g}$ is the DC gate voltage. The first term in Equation (31) denotes the frequency of the graphene mechanical resonator at $V_{g}=0$. The second term represents the spring stiffness hardening effect due to the built-in strain. The third term provides a spring stiffness softening effect due to the nonlinear electrostatic force. The built-in strain plays an important role in controlling the resonant frequency. For small strain, the second term in Equation (31) dominates and the frequency increases monotonically with the gate voltage. For large strain, the third term dominates and the frequency decreases with the gate voltage [102]. For intermediate strain, the resonant frequency firstly decreases and then increases with the gate voltage [103]. All the three cases were experimentally observed by Chen and Hone [53]. In addition, the idea of programming the resonant frequency of an array of resonators was developed theoretically [104] and a multiple state mechanical resonant frequency memory was demonstrated [105].

## 4. Resonator Structures and Materials

Although micro- and nanoresonators have significant applications in many fields, most of these resonators are designed as complex structures that complicate the estimation of their resonant frequencies [106]. The resonant frequency changes should be determined by variations of geometrical variables and mechanical properties of the resonators [107]. Many materials and nanostructures such as SiC [1], carbon nanotubes [31] silicon [32,108,109], graphene [110], Pt [111], GaN [112], rhodium [113] and ZnO [114] were widely used for resonator applications. Micro- and nanomechanical resonators based on the materials, including SiC and group III-nitrides [115], carbon nanotube [54], and graphene sheet [2,52], have been recently reviewed.

### 4.1. Beam/Plate-Type Structure

Micro and nanomechanical resonators of various geometries like cantilever and bridge beams, and plates have found widespread use. The increasing complexity of resonant structure generates challenges in determining the resonant frequency using analytical models except for the experimental measurements [27]. Lobontiu *et al*. [116,117,118] had successfully developed mathematical models to predict the resonant frequency of single and doubly clamped beam resonators with variable cross section or multisegments. Looker and Sader [119] presented an analytical model for the fundamental bending resonant frequency of thin rectangular cantilever plates, which is valid for all aspect and Poisson ratios. Pasini [79] developed an interesting model applied to obtain the resonant frequency of multilayered microresonators with different shaped cross section, symmetry and number of layers, and materials. Considering different loading such as concentrated and uniformly distributed loads, and bending moments acting on the beam at the same time, Herrera-May [106] developed an analytical model for estimating the resonant frequency of micro- and nanoresonators. Zhang *et al*. [120,121,122] demonstrated the effects of tuning on parametrically excited micromechanical resonators. It can be useful in the mechanical design of micro- and nanoresonators with complex structural configurations.

The structural configuration of resonators often contains beam or plate with different cross sections and loading types. Figure 10 in [123] illustrates the characteristic dependence of the resonant frequency on the effective geometric parameter ${\left(t/l^{2}\right)}_{\text{eff}}$ of the doubly clamped beam resonators with different materials. The fundamental out-of-plane and in-plane flexural resonant frequencies of the structure are given by the expressions $\omega_{0}/2\pi =1.03\sqrt{E/\rho }(t/l^{2})$ and $\omega_{0}/2\pi =1.03\sqrt{E/\rho }(w/l^{2})$, respectively. It can be found that the resonant frequency varies linearly with the geometric factor ${\left(t/l^{2}\right)}_{\text{eff}}$ and varies for different materials even the resonator has the same geometric structures. This effect becomes particularly important as the beam size reduces [27]. Greenberg *et al*. [57] also reviewed the dependence of the resonant frequency on the geometric structures and materials of the nanomechanical resonators. With appropriate boundary conditions for a beam of length *L*, Bak *et al*. [124] expressed the length dependence of resonant frequency of a thin-film beam with thickness t and internal stress $\sigma_{\mathrm{int}}$ as [125]:

(32) $$f_{0}=1.03(t/L^{2})\sqrt{E/\rho }\sqrt{1+\sigma_{\mathrm{int}}L^{2}/(3.4Et^{2})}$$

From comparison of different beam lengths for a given width, the resonant frequency $f_{0}$ was verified to be highly dependent on the beam length for compositions both with and without CNTs. Different total beam thickness (50 and 100 nm) leads to different length dependences. For the case of 100 nm resonators of both compositions, Bak *et al* [124] reported that $f_{0}\propto L^{-2}$ for L>~25μm and $f_{0}\propto L^{-1}$ for L<~25μm. The resonant frequency $f_{0}$ can easily be fitted by Equation (33) for 50 nm resonators. For a given geometry, the Al–Al_2_O_3_–CNT (AAC)-nanolaminate-beam resonant frequencies observed surpass those of GaAs, Si and AlN and approach SiC values.

**Figure 10:**
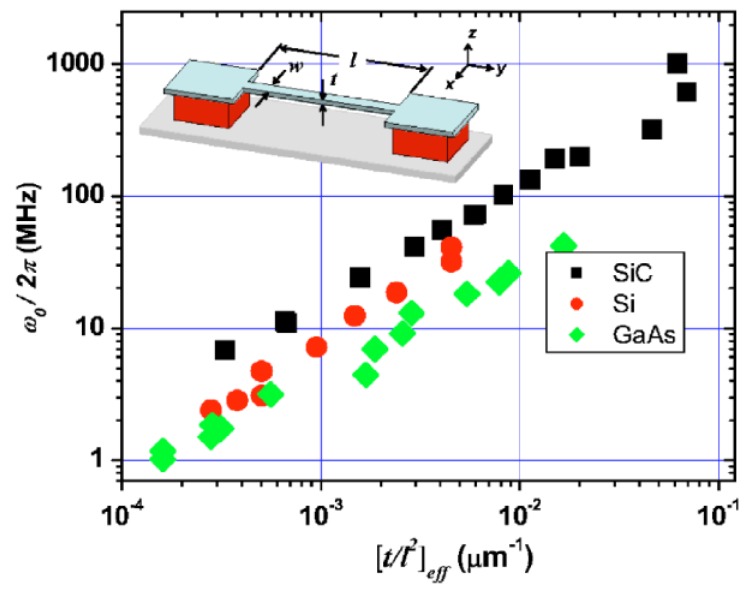
Relationship between the resonant frequency and effective geometry of the doubly clamped beam resonators made from single-crystal SiC, Si, and GaAs. Reused with permission from [123].

Nevertheless, novel architectures dissimilar to the classic beam-like nanomechanical resonators, such as tuning electrode and tuning fork structures and suspended channel and micropillar resonators, have been recently proposed and reported [49,126,127,128]. In the following, we provide the overview of some novel tuning structures and newly micro- and nanomechanical resonators.

### 4.2. Tuning Electrode Structure

**Figure 11:**
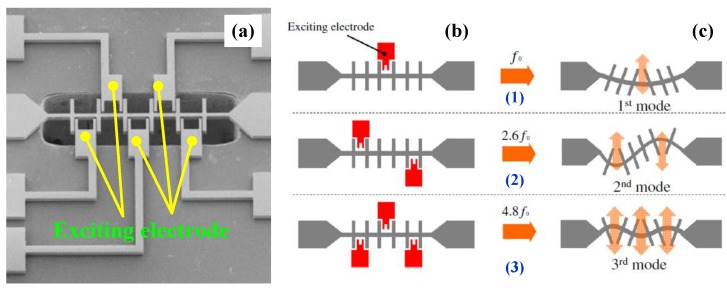
Frequency tuning principle of a fishbone resonator with five exciting electrodes reported by Suzuki *et al*. [129]. (**a**) SEM of a fishbone micromechanical resonator with five exciting electrodes; (**b**) Configurations of the exciting electrodes; (**c**) main beam deformations corresponding to each exciting electrode configuration. Reused with permission from [129].

Suzuki *et al*. [129] designed a fishbone-shaped resonator which has the resonant frequency with a maximum response changes according to the location and number of several exciting electrodes to provide wide-frequency tuning. Furthermore, the selection of tuning frequency among several resonant frequencies was demonstrated [126]. The schematic of the resonant frequency tuning principle is shown in Figure 11, in which the fabricated resonator has six sub-beams formed at the same interval along the main beam and five exciting electrodes. Using these exciting electrodes, the central-electrode (1), trans-electrode (2), and central and cis-electrode (3) configurations can be founded [129]. The resulting frequency tuning covers 178 to 1746 kHz and indicates that the tapered-anchor resonators are suitable for frequency tuning applications. On the other hand, the resonant frequency greatly decreases with the increase of suspension length due to the softening effect on the main beam.

For the first time, Chen *et al*. [28] designed the tuning electrodes underneath pull-in frames to provide a voltage-dependent quasi-linear frequency tuning for CMOS-MEMS resonators. The composite beam resonator is encompassed by the pull-in frames. The resonant frequency can be expressed as [130]:

(33) $$f_{0}^{\text{tun}}=f_{0}\sqrt{1-g(d_{0}(V_{M}),V_{p})}$$

where $f_{0}$ is the pure mechanical resonant frequency of the resonator without electrodes or applied voltages, $d_{0}$ is the electrode-to-resonator gap spacing and controlled by the tuning voltage $V_{M}$, $V_{p}$ is the dc-bias voltage, the function g(•) denotes the effect of an electric stiffness $k_{e}$ to soften the mechanical stiffness. The electrical stiffness $k_{e}$ is capable of modulating the resonant frequency determined by electrode-to-resonator gap spacing. The resonant frequency satisfies $f_{0}=1/(2\pi )\sqrt{(k_{m}-k_{e})/m_{r}}$, where $k_{m}$ and $m_{r}$ are the mechanical stiffness and effective mass of the resonator, respectively. This work had successfully developed the quasi-linear frequency tuning mechanism using the adjustment of modulated bias voltage without consuming any dc power, allowing a 5000-ppm tuning range and a sensitivity of 83.3 ppm/V.

### 4.3. Tuning Fork Structure

Tuning forks are high performance mechanical resonators [131], and typically and widely used as frequency references [132], tunable filters [133], force transducer [134], magnetic field sensing [135]. Although tuning fork structures have been widely investigated and generally modeled by the beam-spring model and two-degrees-of-freedom model [136], the vibration characteristics of nanomechanical tuning forks are not well understood [29,127].

Ashiba *et al*. [127] developed a model of the single-ended tuning forks and used it to predict the resonant frequencies for the in-phase and antiphase modes. The arm of a tuning fork was modeled by a beam connected to one or more torsional springs. Recently, Gronicz *et al*. [137] demonstrated a single-ended tuning fork component with separate signal and tuning electrodes, which make it possible to perform frequency tuning with little interference with the output signal level. The separating tuning and driving electrodes enable the resonant frequency adjustment by over 70,000 ppm.

As one of the typical structures, double-ended-tuning-fork (DETF) can provide high stability, high dynamic range, low mechanical compliance and easily digitizable output signals, and was widely used in mechanical resonators. The DETF structure is composed of two nominally identical suspended parallel tines connected at both ends. Table 2 summarizes some single-ended tuning fork (SETF) and double-ended tuning fork (DETF) structures used for micro- and nanomechanical resonators. Jha *et al*. [138] designed a spring mounted DETF to reduce the axial stress in the beams of the resonator. Agarwal *et al*. [139] found that the increase in resonant frequency is associated with beam length reduction. These scaling rules of nonlinearities in DETF microresonators are useful for the optimization of high precision frequency reference. The asymmetries during fabrication and the mechanical coupling between the tines lead to the frequency separation between the in-phase and the out-of-phase resonant modes [134].

**Table 2:** Overview of some single-ended tuning fork (SETF) and double-ended tuning fork (DETF) resonators.

Component	Fabrication Technique	Frequency Range	Quality Factor	Reference
SETF	silicon-on-insulator wafer by a two-step process	>1.5 MHz	~2000	Gronicz *et al*. [137]
	Focused-ion-beam chemical vapor deposition process	~1.5–11 MHz (10 Pa)	~150–600	Ashiba *et al*. [127]
		~1–11 MHz (0.1 MPa)	~5–50	
DETF	Episeal encapsulation process	~100–2000 kHz	~9000–17,000	Agarwal *et al*. [139]
	Wafer-scale HFCVD diamond deposition process	~0.5–10 MHz	<81,646	Najar *et al*. [140]
	Ge-blade damascene process (GBDP)	24.04 MHz	~6000	Takeuchi *et al*. [141]
	Silicon-on-insulator (SOI) micromachining process	47.2 kHz (in-phase)	26,000	Zhang and Lee [135]
		49.6 kHz (anti-phase)	100,000	
	Silicon on-insulator (SOI) MEMS process	~310 kHz	21,221	Thiruvenkatanathan *et al*. [142]

### 4.4. Graphene Mechanical Resonator

Since the discovery of graphene reported by Novoselov *et al*. [110] in 2004, it has attracted attention due to its unusual two-dimensional structure and wonderful properties such as high Young’s modulus, high resonant frequency and unique electrical behavior. Graphene-based mechanical resonators offer low inertial masses, ultrahigh frequencies, and, in comparison with nanotubes, low-resistance contacts that are essential for matching the impedance of external circuits [143]. The prospects of wide tunability and low dissipation have aroused technical interest in mechanical graphene resonators [144].

Since the graphene is atomically thin, its resonant frequency is dominated by in-plane tension, which can be modified electrostatically by applying a DC voltage. The degree of tunability depends on the initial built-in tension [33,145,146] and can reach 400% with lowest built-in tension [147]. With an applied voltage ranging from 28 V to 26.2 V, the resonant frequency can be tuned from 51.5 MHz to 47 MHz [147]. The large range of strain available in graphene provides opportunity for applications requiring large frequency tuning and high force sensitivity [53]. Although frequency tuning by strain engineering for graphene mechanical resonators have been demonstrated, the deepened and detailed effects of built-in strain on resonator device performances are not well understood and needed to be explored [53,145,148]. For resonant devices with suspended graphene lengths L from ~0.5 to 2 mm, the graphene resonant frequency scales approximately as (1/L), as expected for a thin membrane [148]. The strain of the membrane $\varepsilon_{s}$ varies the resonant frequency significantly and satisfies $f_{0}=1/(2L)\sqrt{E\varepsilon_{s}/\rho }$ [149]. The change of frequency tunability with temperature is due to changes in the tension of the graphene as it is cooled [145]. The thermal expansion of graphene affected the modal dispersion of resonators and reduced the frequency tunability [33]. Exploiting the impermeability of graphene membranes to controllably tune the resonant frequency gives us the mass of the suspended graphene membrane regardless of this initial tension [150]. Because of the remarkable thinness and flexibility of the graphene, the resonant frequency of graphene mechanical resonator can be tuned over a wide range [52]. Bunch *et al*. [150] used pressure differences to tune the mechanical resonant frequency of a monolayer graphene membrane resonator by ~100 MHz. Table 3 summarizes several graphene-based mechanical resonators reported in the literature.

**Table 3:** Comparison of some graphene mechanical resonators reported during the past several years.

Year	Resonator Structure	Excitation Method	Resonant Frequency	Quality Factor
2007	Doubly clamped single layer graphene [151]	Electrostatic/optical excitation	70.5 MHz (room temperature)	78
2008	Fully clamped square graphene [150]	Optical excitation	66 MHz (room temperature)	25
2008	Doubly clamped multilayer graphene [152]	Electrostatic excitation	18–85 MHz	2–30
2008	Fully clamped drum graphene [153]	Optical excitation	10–110 MHz (room temperature)	1500–4000
2009	Doubly clamped single layer graphene [148]	Electrostatic excitation	From 30 to 130 MHz (room temperature)	~100 (room temperature), ~14,000 (at 5 K)
2009	Doubly clamped graphene [154]	Optical excitation	3–100 MHz	50–400
2010	Doubly clamped single layer graphene [145]	Optical/electrical excitation	From 5 to 75 MHz	~250 (room temperature), ~9000 (at 9 K)
2010	Doubly clamped single layer graphene [102]	Electrostatic excitation	34 MHz (at 77 K)	~10,000
2011	Doubly clamped single layer graphene [155]	Electrostatic excitation	~255 MHz	~100,000
2012	Doubly clamped few-layer graphene [156]	Thermal excitation	~8–23 MHZ	~7000
2013	Fully clamped graphene drum resonator [157]	Electrostatic excitation	48–260 MHz	~60
2014	Rectangular membrane graphene resonator [149]	Piezoelectric excitation	15.8–17 kHz	N/A

### 4.5. Suspended Channel Resonator

The most severe limitation of mechanical resonators for sensing applications is their significantly degraded performance in a liquid environment [49,158]. In 2003, Burg *et al*. [30] presented a radically innovation to overcome this limitation using suspended microchannel resonator (SMR) for biosensor applications. During the past decade, further reduction of the size of suspended micro- and nanochannel resonators (SMRs and SNRs) are developed to provide resolution to weigh single viruses and large biomolecules as density and viscosity measurements [159,160,161,162].

**Figure 12:**
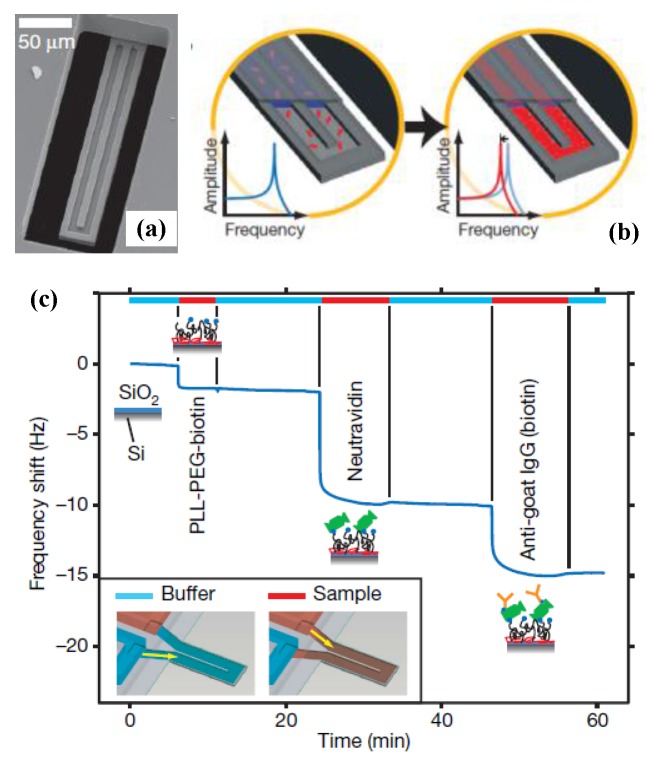
A suspended microchannel microresonator for biomolecular mass sensing reported by Burg *et al*. [163]. Reused with permission from [163], Copyright 2007 Nature Publishing Group. (**a**) Schematic of mass measurement mode by a microcantilever; (**b**) Resonant frequency shifts caused by accumulation of proteins inside the cantilever.

In contrast to the approaches that used an immersed cantilever in fluids, Burg *et al*. [163] developed a SMR which has enabled novel label-free biological sensing applications with unprecedented mass resolution (~1 fg in a 1 Hz bandwidth), as shown in Figure 12. The SMRs are highly sensitive, batch-fabricated microcantilevers with embedded microchannels that can directly quantify adsorbed mass via shifts in resonant frequency, which is position-dependent. The suspended fluid channel constitutes a micromechanical resonator, and the surface adsorption is an effective mechanism for biomolecular mass sensing (Figure 12a). Changes in mass inside the microchannel lead to the resonant frequency shift, both the spring constant $k_{c}$ and the total effective mass $m_{c}$ determine resonant frequency given by:

(34) $$f_{0}=\frac{1}{2\pi }\sqrt{\frac{k_{c}}{m_{c}+\alpha_{c}\Delta m}}$$

where $\alpha_{c}$ is a numerical constant that depends on the geometric localization of the added mass Δm, and $\alpha_{c}\approx 0.24$ for changes in solution density and $\alpha_{c}\approx 1$ when a particle in transit is positioned at the maximum point [163]. The exact mass of the different layers can be quantified by the difference in resonant frequency before and after each injection, as illustrated in Figure 12b. This SMR can weigh single nanoparticles, single bacterial cells and sub-monolayers of adsorbed proteins in water with sub-femtogram resolution. More recently, the SMRs were successfully employed to measure liquid viscosities [164], temperature-dependent density and volume contraction of binary mixtures [165], temperature variations produced by the biological heat sources [166], to handle multiple viscous samples [167] and to examine phase transitions of two materials in liquid state [168]. Table 4 summarizes the comparison of some micro-and nanochannel resonators which were widely applied for density and viscosity measurements. Particularly, the high frequency, high quality factor demonstrated in Table 4 enable strongly localized, high-sensitivity chemical, biological and optomechaical analytes within the fluidic hollow resonators. Modena *et al*. [169] deduced the relationship between the signal-to-noise ratio (SNR) and the limit of detection of suspended micro- and nanochannel resonators as:

(35) $$\text{S/N}\geq \frac{1}{5}\frac{f_{p0}^{2}}{\sigma_{n}^{2}}c_{p}V_{r}\sqrt{T_{\text{meas}}f_{s}}$$

where $f_{p0}$ is the frequency shift induced by a single particle, $c_{p}$ is the average sample concentration, $V_{r}$ is the volume of the resonator, $T_{\text{meas}}$ is the measurement time, $f_{s}$ denotes the sampling rate for an adequately band limited signal, and $\sigma_{n}^{2}$ is the variance of the readout noise and $\sigma_{n}=\begin{array}{cc} 5 & \text{fg} \end{array}$ for the SMR and $\sigma_{n}=\begin{array}{cc} 27 & \text{ag} \end{array}$ for the SNR [169]. Further improvements focus on the design optimization for geometrical dimensions and development of more efficient excitation and detection approaches.

**Table 4:** Overview of some suspended micro- and nanochannel resonators.

Resonator Type	Year	Dimensions of Resonator and Channel (Length × Width × Height)	Actuation/Sensing Method	Resonant Frequency	Quality Factor	Limit of Detection (Sensitivity)	Target/Application	Reference
Microchannel	2003	300μm × N/A × N/A	Electrostatic/optical	~42.7 kHz (air); 40.1 kHz (2-propanol); 39.6 kHz (water)	~90 (air)	${\text{10}}^{\text{-17}}{\text{g/μm}}^{\text{2}}$	Avidin and biotinylated bovine serum albumin	Burg *et al*. [30]
		N/A						
	2006	300 × 33 × 7 μm	Electrostatic/optical	~33.5 kHz	~300–700 (vacuum); ~85 (air)	0.8 ng/cm^2^	Avidin and biotinylated bovine serum albumin	Burg *et al*. [170]
		200 × 33 × 7 μm						
	2007	200 × 33 × 7 μm	Electrostatic/optical	220.5 kHz (air); 209.6 kHz (water)	15,000	0.7 ng/mL	Goat anti-mouse IgG molecules	Burg *et al*. [163]
		N/A × 8 × 3 μm						
	2010	12 × 0.1 × 0.03 mm	N/A	~200 kHz	15,000	10 ng/mL	Activated leukocyte cell adhesion molecule (ALCAM)	von Muhlen *et al*. [171]
	2013	200 × 8 × 3 μm	Optical/optical	2–11,000 MHz	1.6E8	N/A	Water	Bahl *et al*. [172]
	2011	406 × 28.5 × 12 μm	Piezoresistive/optical	92.1 kHz	10,850	18.1 fg	Budding yeast cells	Lee *et al*. [173]
		N/A × 7.9 × 8 μm						
	2013	200 × 20 × N/A μm	Piezoresistive/optical	~137.7 kHz	~15,000	16 Hz/kg/m^3^	very light solvents to very viscous and sticky crude oil samples	Khan *et al*. [167]
		N/A × 4 × 3 μm						
	2014	60 × 36 × 7 μm	Feedback loop/optical	1.17 MHz	~23,000 (gas); ~6000 (liquid)	~30 fg	polystyrene nanoparticles	Modena *et al*. [169]
		N/A × 8 × 3 μm						
	2013	300 × 30 × 30 μm	Thermal/optical	2.21 MHz (ambient atmosphere); 1.25 MHz (water)	190 (ambient atmosphere); 170 (water)	8.6 ppm/μW	Biological molecules and individual cells	Toda *et al*. [166]
Nanochannel	2010	20 μm × 650 nm~2.5 μm × 107 nm	Thermal/optical	~25 kHz	1300–7000 (before filling)	2 fg	Ethanol, H_2_O and D_2_O	Barton *et al*. [161]
	2010	50 × 10 × 1.3 μm	Electrostatic/optical	~630 kHz	~8000	27 ag	Ethanol, H_2_O and D_2_O	Lee *et al*. [174]
		N/A × 2 μm × 700 nm						

## 5. Major Influencing Factors

In this section, we briefly review the major influencing factors associated with making tunable micro- and nanomechanical resonators and describe the efforts implemented to predict, control and apply the resonant frequency shift for overcoming challenges and extending applications.

### 5.1. Large-Amplitude Effect

In the field of micro- and nanomechanical resonator design, it is a common misconception that large-amplitude motion [175]. The high-amplitude operation of micro- and nanomechanical resonators may be useful for various reasons, such as achieving a suitable SNR, and is suitable for signal processing, mass and force sensing, micro-gyroscope applications [176,177,178], and the development of other new technologies [179,180]. However, it is difficult to enter into the large-amplitude regime for a traditional mechanical system because the dynamic range of the system decreases dramatically as the dimensions of the resonator are reduced [181,182]. In the derivation of of Equation (1), small amplitudes of the vibration $w_{\max}$ were assumed. To make a clear understanding of the large-amplitude effect on the resonant frequency, an additional potential energy term from stretching of the mid-plane of the beam is included in Rayleigh’s quotient. The modified expression can be written as [183]:

(36) $$\omega_{n,N}(w_{\max})=\omega_{n,N}(0){\left(1+\gamma_{n}\frac{NL^{2}}{12EI}+\alpha_{n}\frac{EA}{12EI}w_{\max}^{2}\right)}^{1/2}$$

where $\omega_{n,N}(0)$ is the resonant frequency of mode *n* for zero axial load and ignoring nonlinear large-amplitude effects, $w_{\max}$ is the amplitude of the vibration and $\alpha_{n}$ is a constant given by:

(37) $$\alpha_{n}=\frac{3}{\phi_{n,\max}^{2}}\left[{\left(\int_{0}^{L}\frac{d\phi_{n}(x)}{dx}\right)}^{4}dx/{\left(\int_{0}^{L}\frac{d^{2}\phi_{n}(x)}{dx^{2}}\right)}^{2}dx\right]$$

where $\phi_{n,\max}$ is the maximum value of the approximate shape function $\phi_{n}$.

Nonlinear large-amplitude vibration has important role in micro- and nanomechanical resonators [151,184], which often are driven into non-linear regime with larger amplitude in order to store enough energy [56]. Although practical relevance of large-amplitude effect on the resonant frequency of resonators has been reported, up to now, a few investigations discussed the large-amplitude regime. Recently, Bagheri *et al*. [182] firstly demonstrated the zero frequency singularity in nanomechanical resonators in the large-amplitude regime, which provides a new mechanism to tune the resonant frequency of the resonator over a large range.

### 5.2. Surface Stress Effect

**Figure 13:**
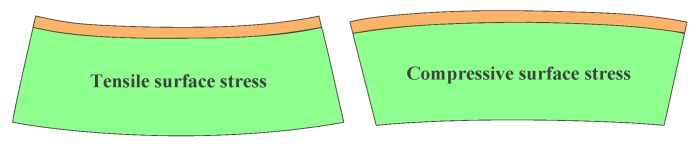
Schematic of the tensile surface stress (positive surface stress) inducing a concave curvature and the compressive surface stress (negative surface stress) inducing a convex curvature.

Surface stress has a great effect on the mechanical and physical properties of materials and devices. The origin of surface stress can be understood from the chemical bonding of atoms at the surface [185], as shown in Figure 13. Generally, the influence of surfaces can be described either by surface energy or surface stress. The surface stress $\sigma_{ij}$, which is defined in term of surface energy γ, can be described [186] as:

(38) $$\sigma_{ij}=\gamma \delta_{ij}+\partial \gamma /\partial \varepsilon_{ij}$$

where $\delta_{ij}$ is the Kronecker delta and $\varepsilon_{ij}$ is the surface strain tensor. Mathematically, the surface stress $\sigma_{\tau }$ is decomposed into residual (strain-independent) and surface elastic (strain-dependent) terms as [187,188]:

(39) $$\sigma_{\tau }=\tau_{0}+C_{0}\varepsilon$$

where $\tau_{0}$ and $C_{0}\varepsilon$ are the residual (strain-independent) and surface-elastic (strain-dependent) parts of the surface stress, respectively, and where $C_{0}$ is the surface elastic stiffness and $C_{0}=\partial \sigma_{\tau }/\partial \varepsilon {|}_{\varepsilon =0}$. For the one-dimensional and linear case, $C_{0}$ denotes the surface Young’s modulus $E_{s}$ [189].

**Figure 14:**
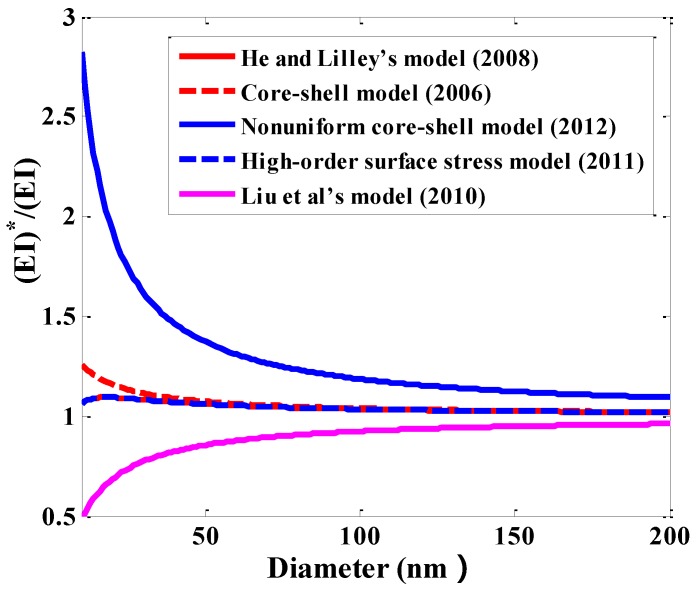
Comparisons of normalized effective bending stiffness in circular NWs calculated by different surface elasticity models with surface layer thickness 1 nm.

Table 5 provides the comparison of some surface elastic models for surface Young’s modulus reported in the literature. Figure 14 shows the comparisons of normalized effective bending stiffness of circular NWs using some surface elasticity models listed in Table 5. For a circular NW, the expression of the effective bending stiffness calculated by high-order surface stress model [190] after incorporating surface moment remains the same as that by He and Lilley [191].There exists obvious difference of bending stiffness in NWs between He and Lilley’s model [191], core-shell model [192,193] and the non-uniform core-shell model developed by Yao *et al.* [194].

**Table 5:** Comparison of some surface elastic models for surface Young’s modulus of nanowires resonators (The relative parameter descriptions can be seen in the literature).

Model	Formulation	Materials	Theory and Method	Effects
Surface elasticity model [195]	${\left(EI\right)}^{\text{*}}=EI+2E^{s}wh^{2}$	GaAs	Classical beam theory	Surface elasticity
Surface Cauchy-Born (SCB) model [196]	$c_{IJKL}^{s}=M_{IJKL}^{s}-A_{IJp}^{s}A_{KLq}^{s}{\left(D^{-1}\right)}_{pq}^{s}$	Si	Based on standard bulk Cauchy-Born model	Surface stress
He’s model [191]	${\left(EI\right)}^{*}=\{\begin{array}{ll} EI+E_{s}wh^{2}/2+E_{s}h^{3}/6 & \text{(rectangle)}\\ EI+\pi E_{s}{d_{c}}^{3}/8 & \text{(circular)} \end{array}$	Al and Si	Euler-Bernoulli beam theory and Young-Laplace equation	Surface stress with different boundary conditions
High-order surface stress model [190]	${\left(EI\right)}^{*}=\{\begin{array}{ll} EI+E^{s}wh^{2}/2+E^{s}h^{3}/6+E^{s}whh_{1}+8D^{s}w & \text{(rectangle)}\\ EI+\pi E^{s}d^{3}\text{/8} & \text{(circular)} \end{array}$	Si	Generalized Young–Laplace equation	High-order surface stress and surface moment
Liu’s model [197]	${\left(EI\right)}^{*}=\{\begin{array}{cc} EI+\left(2\mu_{0}\text{+}\lambda_{0}\right)\left(2bh^{2}+4h^{3}/3\right)-2\upsilon I\tau_{0}/H & \text{(rectangle)}\\ EI+\pi D^{3}\left(2\mu_{0}\text{+}\lambda_{0}\right)/8-2\upsilon I\tau_{0}/H & \text{(circular)} \end{array}$	Al and Si	Gurtin–Murdoch theory	Surface stress, surface elasticity and surface density
Core-shell model [192,193]	${\left(EI\right)}^{*}=\{\begin{array}{ll} Ew_{c}{h_{c}}^{3}/12+E_{s}tw_{c}{(h_{c}\text{+2t)}}^{2}+2E_{s}t{\left(h_{c}/2+t\right)}^{3}/3-2E_{s}t^{3}(w_{c}+2t)/3 & \text{(rectangle)}\\ \pi (E-E_{s}){d_{c}}^{4}/64+\pi E_{s}{\left(d_{c}+2t\right)}^{4}\text{/64} & \text{(circular)} \end{array}$	ZnO	resonance experiment and linear surface elastic theory	Surface layer thickness
Rudd’s model [198]	$E_{tot}=E_{core}[1+(C_{tot}t/A_{tot})\Delta ]+O(t^{2}/R^{2})$	hydrogen-passivated Si	First-principles density functional theory	Plane-wave cut-off energy
Feng’s model [189]	$E^{*}=\frac{4\sqrt{A}\tau_{0}(E_{0}t+4E_{s})}{\sqrt{A}(E_{0}tl+4E_{s}l+4t\tau_{0}+6l\tau_{0})-4(E_{0}t+4E_{s})\tanh(\sqrt{A}l/4)}\left(\frac{t}{l+t}\right)$	nanoporous materials	Gurtin–Murdoch theory	Surface energy and residual surface stress
Yan’s model [199]	${\left(EI\right)}^{*}=\{\begin{array}{ll} \frac{1}{12}(c_{11}+e_{31}^{2}/\kappa_{33})bh^{3}+(c_{11}^{s}+e_{31}^{s}e_{31}/\kappa_{33})(bh^{2}/2+h^{3}/6) & \text{(rectangle)}\\ \frac{\pi D^{4}}{64}(c_{11}+e_{31}^{2}/\kappa_{33})+\frac{\pi D^{3}}{8}(c_{11}^{s}+e_{31}^{s}e_{31}/\kappa_{33}) & \text{(circular)} \end{array}$	piezoelectric materials	Generalized Young–Laplace equations	residual surface stress, Surface elasticity and piezoelectricity
Non-uniform core-shell model [194]	$\begin{array}{l} {\left(EI\right)}^{*}=\pi E\{\frac{D^{4}}{64}{\left(1-2r_{s}/D\right)}^{4}+\frac{D^{3}r_{s}}{8\alpha_{0}}[e^{\alpha_{0}}-{\left(1-2r_{s}/D\right)}^{3}]\\ -\frac{3D^{2}r_{s}^{2}}{4\alpha_{0}^{2}}[e^{\alpha_{0}}-{\left(1-2r_{s}/D\right)}^{2}]+\frac{3Dr_{s}^{3}}{\alpha_{0}^{3}}[e^{\alpha_{0}}-(1-2r_{s}/D)]-\frac{6r_{s}^{4}}{\alpha_{0}^{4}}(e^{\alpha_{0}}-1)\} \end{array}$	ZnO	Nonlinear surface elastic theory	Non-uniform surface elasticity

#### 5.2.1. Axial Stress/Strain Effect

Many resonators are operated under tension, which likely results from the fabrication process and increase the resonant frequency [151]. When the axial load N is taken into account in the resonator, its lateral displacement caused by vibration is w(x,t). The governing equation can be given by [20,181]:

(40) $$EI\frac{\partial^{4}w(x,t)}{\partial x^{4}}+\rho A\frac{\partial^{2}w(x,t)}{\partial t^{2}}-\frac{\partial w}{\partial x}\left[N(x)\frac{\partial w(x,t)}{\partial x}\right]=0$$

where N(x) the actual axial loading and consists of the following two parts:

(41) $$N(x)=N_{0}+\frac{EA}{2L}{\int_{0}^{L}\left(\frac{\partial w(x,t)}{\partial x}\right)}^{2}dx$$

where $N_{0}$ is the axial loading depending on the built-in strain [15], fabrication process [200], residual stress [201], temperature [202], and surface stress [203]. The second part is the tension due to nonlinear mid-plane stretching [15,204]. The resonant frequency can be obtained by employing Rayleigh’s energy method as [183]:

(42) $$\omega_{n,N}=2\pi f_{n,N}\approx \omega_{n}{\left(1+\gamma_{n}\frac{NL^{2}}{12EI}\right)}^{1/2}$$

where $\gamma_{n}$ is a mode-dependent coefficient and satisfies $\gamma_{n}=12/L^{2}\left[{\left(\int_{0}^{L}d\phi_{n}(x)/dx\right)}^{2}dx/{\left(\int_{0}^{L}d^{2}\phi_{n}(x)/dx^{2}\right)}^{2}dx\right]$, in which $\phi_{n}(x)$ represents an approximate shape function for a particular mode n. For n≥3, $\gamma_{n}=0.2949,0.1453,0.\partial 812,\ldots$ [107]. It can be found from Equation (42) that the tensile axial force can lead to an increase of the resonant frequency of the beam, which is known as the “hard-spring effect” [183]. The overestimation of $\omega_{n,N}$ can increase up to 11.6% for high axial strains or for intermediate strains but high length-to-width ratios [205]. Under the action of a large tension N $\beta_{T}=\sqrt{EI/NL^{2}}\ll 1$), the resonance frequency can be expressed as [202,206]:

(43) $$f_{N}=\frac{n}{2L}\sqrt{\frac{N}{\rho A}}\left[1+2\beta_{T}+\left(4+\frac{\pi^{2}n^{2}}{2}\right){\beta_{T}}^{2}\right]$$

By $\varepsilon_{s}=\sigma /E=N/(EA)$, the axial strain $\varepsilon_{s}$ can easily be related to the axial stress σ or the axial force *N*. The expression of resonant frequency can be re-written as [207]:

(44) $$f_{N}=f_{0}\sqrt{1+N/N_{cr}}$$

where $N_{cr}$ is the critical buckling load for a beam, and $N_{cr}=7EAh^{2}/(2L^{2})$ for the cantilever [20] and $N_{cr}=4\pi^{2}EI/2L^{2}$ for clamped-clamped beam [207]. Karabalin *et al*. [203] successfully presented the expressions for the relative frequency shifts $\Delta f/f_{0}$ of cantilever and doubly clamped beam resonators due the effect of surface stress and geometric effect, which is typically ignored in the classical theory of linear elasticity, as listed in Table 6. The application of surface stress induces the change in the beam length, width, thickness, and density, which alter the resonant frequency of the beam resonators. It can be found that the resonant frequencies of doubly clamped beams are more sensitive to surface stress changes than cantilever beams. The application of the resonant frequency shift expressions can also enable the axial force to be calibrated against the applied voltage.

**Table 6:** Relative frequency shift of cantilever and doubly clamped beam resonators with surface stress and geometric effects reported by Karabalin *et al*. [203]. A normalized load $\bar{\sigma }=(1-\nu )\sigma_{s}^{T}/(Eh)$ is applied and $\sigma_{s}^{T}$ is an applied surface stress.

Resonator Structure	Relative Frequency Shift $\Delta f/f_{0}$	
	Stress Effect	Geometric Effect
Cantilever beam	$-0.042\nu (b/L){\left(b/h\right)}^{2}\bar{\sigma }$	$[(1+2\nu )/(1-\nu )]\bar{\sigma }$
Doubly clamped beam	$0.1475{\left(L/h\right)}^{2}\bar{\sigma }$	$\{(1+2\nu )(1+\nu )/[2(1-\nu )]\}\bar{\sigma }$

For the case of SWCNT, the resonant frequency related to the axial strain can be written as [207]:

(45) $$f_{N}=f_{0}\sqrt{1+\frac{2L^{2}}{\pi^{2}(4R_{0}^{2}+t_{0}^{2})}\varepsilon_{s}}$$

where $R_{0}$ depends on the SWCNT chirality, $t_{0}$ is the fixed thickness of the hollow beam section, and $\varepsilon_{s}$ is the axial strain and its sign or direction can result in the resonant frequency becoming either greater or less than the un-tuned one. The frequency shift varies linearly with nano-strain when $\varepsilon_{s}$ is very small. The resonant frequency itself may be tuned by increasing tensile strain, which can be expressed for the oscillation-induced effective strain as $\varepsilon_{s}=(3/4)\pi^{2}\alpha_{s}E_{k}^{0}/(m\omega_{0}^{2}L^{2})$, where $\alpha_{s}$ is the actuation energy parameter, $E_{k}^{0}$ is the total kinetic energy [208]. It provides a direct and useful relationship between the applied mechanical tensile strain and the strain induced by nonlinear oscillations to the resonant frequency of the resonator. For the inadequate strain, Chaste *et al*. [209] presented contracting Au electrodes upon cooling to increase the tensile stress within the CNT and tune the resonance frequency less than 100%. The resonant frequencies are about ten times higher than those without taking into account the tensile stress within the SiNx layer [210]. Tensile stress in SiC resonators [211] caused an increase in resonant frequency of more than 100%.

**Figure 15:**
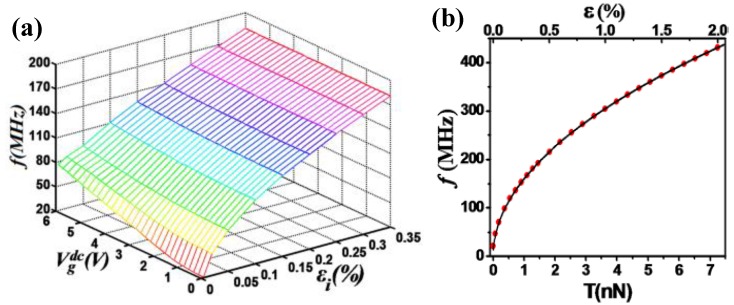
The change of resonant frequency of the CNT as a function of the gate voltage and the axial strain (**a**) and the tension (**b**) reported by Ning *et al*. Reused with permission from [212].

More recently, Ning *et al*. [212] reported a new design of carbon nanotube (CNT) resonator, whose resonance frequency can be tuned not only transversally by a gate voltage, but also by the axial strain applied through directly pulling the CNT, as shown in Figure 15. It can be seen from Figure 15a that the gate-tuning ability decreases as the strain increases. The resonant frequency increases up from 9.44 MHz to 21.04 MHz with only 0.004% strain, as shown in Figure 15b, indicating the super sensitivity of the resonator to strain. When the the CNT with 2% strain resulting in the tension to about 7.24 nN, while the gate-induced tension is usually less than 0.5 nN [31], the resonant frequency can increase by more than twenty times than that in gate-induced nanotube resonators [69,213,214,215]. Large stress levels developed during the insulator-to-metal transition can result in the frequency tuning for about 23% and the large sensitivity of buckled microbridge resonators is attributed to the stress changes [216].

#### 5.2.2. Residual Surface Stress

**Figure 16:**
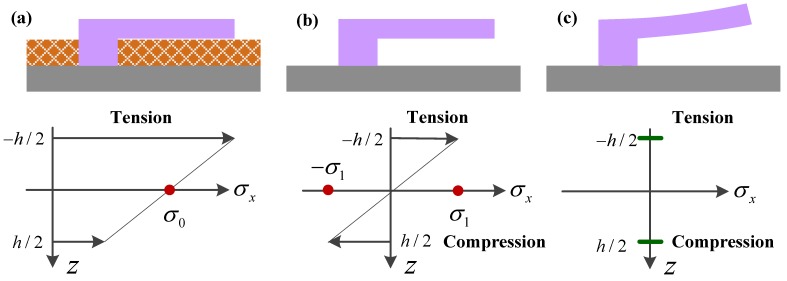
Schematic of residual stress gradient in the cantilever with microfabrication. (**a**) An ideal intermediate state, (**b**) after release and (**c**) before bending. Reused with permission from [224], Copyright 2010, John Wiley and Sons.

The origin of residual stress can be attributed to the mismatch of both thermal expansion coefficient and intrinsic stress due to the microstructure of the film, grain size, variation of growth rate during film deposition [217,218]. Residual stress is unavoidable in surface micromachining techniques and is difficult to control since the fabrication process involves many temperature cycles [219], and can be given by:

(46) $$\sigma_{r}=\sigma_{0}(1-\nu )$$

where $\sigma_{0}$ is the biaxial residual. In most designs, the residual stress due to the microfabrication process is highly undesirable and is released within the film, as shown in Figure 16. The surface stress contains residual (strain-independent) and surface elastic (strain-dependent) terms. It is difficult to identify which parts of the surface stress in the formulation actually affect the resonant frequency of the resonators. On one hand, using the linear elastic continuum theory, Gurtin *et al*. [195] demonstrated that the resonant frequency is independent of the strain-independent surface stress *τ_0_*. Lu *et al*. [220] reported that the resonant frequency is only influenced by the strain-dependent part of the surface stress. On the other hand, Lachut and Sader [221] found that the effect of the strain-independent part of the surface stress on the resonant frequency can be obtained using fully three-dimensional models. Effects of residual and axial stresses on the micro-beam resonators were reported in [222]. Park and Klein [188,223] quantified, for the first time, how both the residual (strain-independent) and surface elastic (strain-dependent) parts of the surface stress impact the resonant frequencies of metal nanowires.

Lachut and Sader [221] predicted the resonant frequency shift due to the strain-independent surface stress within the context of linear elastic beam theory and obtained:

(47) $$\frac{\Delta f}{f_{0}}=-0.042\frac{\nu (1-\nu )\sigma_{s}}{EL}$$

where $\sigma_{s}$ is the strain-independent surface stress. It indicates that the frequency shift due to the strain-independent surface stress should be dependent only on the nanowire length [221].

#### 5.2.3. Adsorption-Induced Surface Stress

Surface stress induced by adsorption can play very important role in the resonant frequency [225,226]. Various models have been proposed to explain adsorption-induced surface stress effect on the resonant frequency shift of a resonator in vacuum or in gaseous environment [227,228,229]. The surface stress effect can be modeled as an axial force exerted on the resonant structures using two different models, including the concentrated load mode [230,231,232,233] and the distributed load model [234]. Determining the adsorption-induced surface stress and mass from the experimental data of resonant frequencies becomes an inverse problem [234,235].

To explain the adsorption on the stiffness effect, the model presented by Ramos *et al*. [90,95] is a representative example. The cantilever was modeled as an Euler-Bernoulli beam and differential equation of the vibration can be written as:

(48) $$\left(\rho A+\lambda (x)\right)\frac{\partial^{2}w(x,t)}{\partial t^{2}}+\frac{\partial^{2}}{\partial x^{2}}\left[D(x)\frac{\partial^{2}w(x,t)}{\partial x^{2}}\right]=0$$

where λ(x) is the adsorbed mass per unit length, D(x) is the flexural rigidity of the cantilever and is expressed as:

(49) $$D(x)=\frac{b}{12}\frac{E_{c}^{2}T_{c}^{4}+E_{b}^{2}T_{b}^{4}(x)+E_{c}E_{b}T_{c}T_{b}(x)[2T_{c}^{2}+2T_{b}^{2}(x)+3T_{c}T_{b}(x)]}{E_{c}T_{c}+E_{b}T_{b}(x)}$$

where *b* is the cantilever width, *T* is the thickness, and *E* is the Young’s modulus, the subscripts *c* and *b* represents the cantilever and the bacteria, respectively. The resonant frequency is calculated by the Rayleigh approximation method as [90]:

(50) $$\omega_{n,N}=2\pi f_{n,N}=\frac{\int_{0}^{L}D(x){\ddot{\psi }}_{n}^{2}(x)dx}{\int_{0}^{L}\left[\rho A+\lambda (x)\right]\psi_{n}^{2}(x)dx}$$

where $\psi_{n}(x)$ is the eigenmode shape of the unloaded cantilever when the average rates of kinetic energy and potential energy equals and the transverse vibration is assumed to satisfy $w(x,t)=A_{r}\psi_{n}(x)\cos(\omega_{n}t+\phi )$, in which $A_{r}$ and ϕ are the arbitrary values of the amplitude and phase of the vibration. The equality of the energies explains that the resonant frequency decreases when a mass is added to the resonator [88].

**Figure 17:**
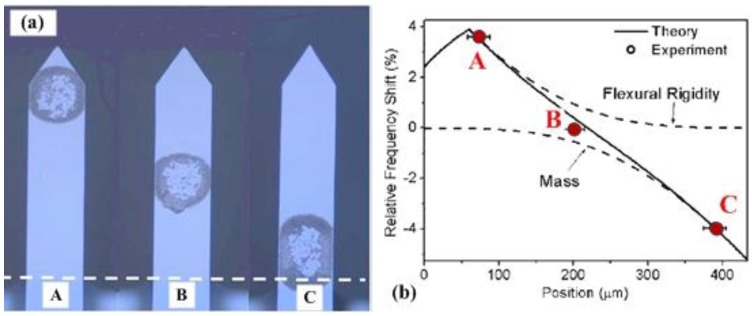
(**a**) Optical micrographs of three silicon cantilevers in which bacteria deposited near the free end (A), on the middle (B), and near the clamping (C) at three different positions; (**b**) Resonant frequency shift as a function of the longitudinal position of the adsorbed bacteria with respect to the clamping. Reused with permission from [90].

Figure 17 shows experimental and calculated frequency shift *versus* the adsorption position along the cantilever due to the added mass (dashed line), the change of flexural rigidity (dashed line), and both effects (solid line). The interesting results indicate that the adsorbed bacteria can induce the positive resonant frequency shift in nanomechanical resonators for certain adsorption distributions on the cantilever. An extensive equation for the resonant frequency shift due to the adsorption of a homogeneous layer on the cantilever that explains the effects of added mass and stiffness of the layer can be written as [88]:

(51) $$\frac{\Delta f_{n,N}}{f_{n,N}}≅\frac{1}{2}\left(3\frac{E_{b}}{E_{c}}-\frac{\rho_{b}}{\rho_{c}}\right)\left(\frac{T_{b}}{T_{c}}\right)+\frac{3}{8}\left[{\left(\frac{\rho_{b}}{\rho_{c}}\right)}^{2}+2\frac{E_{b}}{E_{c}}\left(4-\frac{\rho_{b}}{\rho_{c}}\right)-7{\left(\frac{E_{b}}{E_{c}}\right)}^{2}\right]{\left(\frac{T_{b}}{T_{c}}\right)}^{2}$$

where *ρ* is the density. The adsorption-induced shift of the resonant frequency of the nanomechanical resonators due to three effects, including added mass (negative), mechanical stiffness (positive), and surface stress (negative or positive) are reviewed and discussed in [236] for biosensor applications. However, the mechanical properties of adsorbed molecules became increasingly important as the size of the resonator reduces. The adsorption-induced frequency shift can be enhanced by increasing the actuation energy [208]. Goeders *et al*. [237] presented a most general case of the change in resonant frequency due to adsorbed mass (Δ*m*), to increased stiffness Δ*k* due to a change in chickness, and to surface stress (Δ*σ*) as:

(52) $${f_{n}}^{\Delta m,\Delta k,\Delta \sigma }=\frac{{\left(\alpha_{n}^{\Delta \sigma }\right)}^{2}}{2\pi \sqrt{3}}\sqrt{\frac{k}{m_{0}+\Delta m}+\frac{3E_{\text{ads}}I_{\text{ads}}}{L^{3}(m_{0}+\Delta m)}}$$

where $\alpha_{n}^{\Delta \sigma }=\alpha_{n}{\left(1+2\sigma L^{3}/(\pi^{2}EI^{3})\right)}^{1/4}$, $E_{\text{ads}}$ and $I_{\text{ads}}$ are the elastic modulus and second moment of the adsorbed layer, respectively.

#### 5.2.4. Fluid Effect

In particular, micro- and nanomechanical resonators have widely used in gas, air or liquid environment, the viscous damping resulting from viscous fluids strongly affects resonant frequency responses of resonators and the fluid-structure interaction on the resonant frequency makes it challenging to perform measurement in viscous fluids [66,81,238,239]. A more rigorous approach, which was first introduced by Sader [66], is widely used to investigate of the frequency response of immersed cantilevers under the hydrodynamic forces due to the surrounding fluid. The fluid flow around the microcantilever can be governed by the incompressible Navier-Stokes equations as:

(53) $$\rho_{f}\frac{\partial v}{\partial t}+(v•\nabla )v=-\nabla p+\eta_{f}\nabla^{2}v$$

where v is velocity field, *p* is the pressure, $\rho_{f}$ and $\eta_{f}$ are the density and viscosity of the fluid, respectively. The inviscid model can be applied to accurately predict the resonant frequency in fluids. The deviation from the inviscid model can be addressed using the Reynolds number Re arguments [66]. The Reynolds number Re for a cantilever beam vibrating in a viscous fluid neglecting the nonlinear inertial terms of the Navier-Stokes equation is given by:

(54) $$Re=\frac{\rho_{f}\omega b^{2}}{4\eta_{f}}$$

where $\rho_{f}$ is the density of the fluid, *ω* is the angular frequency, and $\eta_{f}$ is the viscosity of the fluid. For the practical cases Re≫1, the inviscid fluid model is applicable. However, when the cantilever width is reduced, the Reynolds number Re decreases and the dimensionless hydrodynamic function can be used to account for the viscous effect.

**Figure 18:**
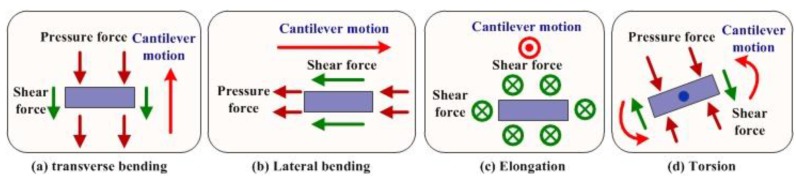
Schematic of the hydrodynamic force acting on the microcantilever cross section in the cases of four modes. (**a**) Transverse (out-of-plane) bending mode; (**b**) Lateral (in-plane) bending mode; (**c**) Elongation (in-plane) mode; (**d**) Torsional (out-of-plane) mode.

It is worth mentioning that Sader [66] presented an accurate theoretical model to predict the hydrodynamic effects on the resonant behaviors of cantilever beam immersed in the viscous fluid. Afterwards, some analytical and theoretical models [238,239,240,241,242], experimental measurements [243,244,245] and modeling and simulations [240,246,247] have been reported on the hydrodynamic effects. The equation of motion for the Sader’s model with hydrodynamic effect can be written as [66]:

(55) $$EI\frac{\partial^{4}w(x,t)}{\partial x^{4}}+\rho A\frac{\partial^{2}w(x,t)}{\partial t^{2}}=f_{hydro}(x,t)+f_{drive}(x,t)$$

where $f_{hydro}(x,t)$ is the hydrodynamic force due to the motion of the fluid around the beam, and $f_{drive}(x,t)$ is the driving force that excites the beam. According to the vibration mode and on the cantilever surface [240], the total hydrodynamic force includes both the effects of the pressure (normal to the surface) and shear stress (tangential to the surface) exerted by the fluid, as shown in Figure 18. The surrounding fluid acts on the cantilever when the additional mass is attached to the cantilever [248]. The *n*th order resonant frequency in the fluid with small dissipative effects can be given by [66,81]:

(56) $$f_{n,f}=\{\begin{array}{cc} f_{n}{\left(1+\frac{\pi \rho_{f}b}{4\rho h}\Gamma_{r}(f_{n,f})\right)}^{-1/2} & \left(\text{flexural mode}\right)\\ f_{n}{\left(1+\frac{3\pi \rho_{f}b}{2\rho h}\Gamma_{t}(f_{n,f})\right)}^{-1/2} & \text{(torsional mode)} \end{array}$$

where $\Gamma_{r}(f_{n,f})$ and $\Gamma_{t}(f_{n,f})$ are the real part of the appropriate hydrodynamic functions for the given geometry, and are calculated and compared for flexural and torsional modes by Van Eysden and Sader [81]. Many researchers had presented theoretical models and experimental measurements to predict the hydrodynamic functions. In the work of Sader [66], hydrodynamic function was described by the complex Bessel functions which have not provided a direct insight into the relationship between the cantilever vibration and the viscous fluids. Green and Sader [249] derived an elegant semi-analytical model to predict the hydrodynamic functions for microcantilevers in viscous fluids. However, Maali *et al*. [250] experimentally verified the model [249] suffers from the same limitations as in [66] showing that the errors in estimation of the damping and added mass coefficients. In addition, an analytical approximation was developed to analyze the hydrodynamic function for the range of Re between 1 and 1000. In the Maali’s expression, $\Gamma_{r}$ can be written as $\Gamma_{r}=a_{1}+a_{2}/\sqrt{2Re}$, in which $a_{1}=1.0553$ and $a_{2}=2.6868$ $a_{1}=1.0553$ and $a_{2}=3.7997$ are empirical parameters determined by fitting the experimental data [245] to the exact hydrodynamic function on Sader [66]. The relative resonant frequency can be obtained as $f_{n,f}=f_{n}{\left[1+\pi \rho_{f}b/(4\rho h)\left(a_{1}+a_{2}\sqrt{\eta_{f}f_{n,f}/(\pi \rho_{f}b^{2})}\right)\right]}^{-1/2}$ [245]. An overview of the relevant hydrodynamic force equations for three different modes of vibration (transverse bending, lateral bending and elon-gation) was reported in [240]. Basak *et al*. [251] developed a fully three-dimensional finite element-based fluid structure interaction model to predict the hydrodynamic loading of microcantilevers in viscous fluids. The hydrodynamic force acting on the beam can also be evaluated by accounting for both the shear force and the pressure force [242]. To facilitate computation for arbitrary *κ*, Eysden and Sader [81] presented an accurate formulas for the hydrodynamic functions that may be used in place of the exact analytical solutions, and the maximum error is 0.6% from the accuracy of approximant representation. The corresponding formulas for the flexural and torsional modes become [81]:

(57) $$\{\begin{array}{l} \Gamma_{r}(\kappa )=\frac{1+0.74273\kappa +0.14862\kappa^{2}}{1+0.74273\kappa +0.35004\kappa^{2}+0.058364\kappa^{3}}\\ \Gamma_{t}(\kappa )=\frac{1}{16}\left(\frac{1+0.37922\kappa +0.072912\kappa^{2}}{1+0.37922\kappa +0.088056\kappa^{2}+0.010737\kappa^{3}}\right) \end{array}$$

It is important to note that the normalized mode number κ, which differs from the actual mode number *n*, affects the hydrodynamic function due to the fluid effect [81]. The Reynolds number Re of nano- and micro- cantilevers are very low, in the range of 10^−2^~10^2^. A few reports have experimentally characterized the Reynolds number Re to interpret fluid effects on the resonant frequency [25]. Cranch *et al*. [252] presented the rigorous experimental validation of the theoretical model [253] for the displacements of both planar and cylindrical cantilevers in highly viscous fluids over a broad range of Reynolds number covering $4\times 10^{-3}\leq Re\leq 2000$ at frequencies up to 1 kHz. One promising approach to reduce the viscous damping for resonators immersed in a fluid is the use of vibration modes which are less affected by the fluid like in-plane flexural bending modes [254,255]. Linden *et al*. [256] observed the resonant frequency shift of 44 kHz caused solely by the adhesion of the latex bead with a mass (550 pg) for the plate-microresonator operated in liquids. The resonant frequency of the cantilever beam immersed in a viscous fluid is critically dependent on the fluid viscosity. The resonant frequency of a microcantilever operating in a fluid is roughly proportional to the inverse of its length squared, and it increases by ~23% with decreasing the length by 10% [242]. Sawano *et al*. [26] achieved mass measurement of biological molecules in viscous fluids using carbon nanotube resonators subject to temperature changes in the environment. The resonant frequency of the nanotube resonators in water decreases with lowering the water temperature. The resonant frequency shift induced by molecular interactions attributes to not only the mass of molecules involved in molecular interactions but also the hydrodynamic effect arising from the hydrophilicity change during the interactions. In addition, temperature dependence of the resonant frequency of resonators in vacuum or in gaseous environments have also been extensively investigated theoretically as well as experimentally [77,138].

Nevertheless, nonlinearities occur at very low amplitudes may greatly reduce the dynamic range of micro- and nanomechanical resonators and hence the device performances. To make a breakthrough of this limitation and overcome frequency stability issues in MEMS and NEMS resonators, some improvements had been performed concerning the part dealing with nonlinear dynamics [111], frequency stability [257] and how to overcome these limitations using nonlinearities cancellation [258,259], superharmonic resonance [260] and simultaneous resonances [261,262]. A theoretical model presented by Kacem *et al*. [258] was a quick and powerful tool for optimizing frequency and nonlinearity tuning as well as to give the possibility to drive the resonator beyond its critical amplitude and enhance the performance of NEMS-based sensor. The real specificities come from the complex nonlinear dynamics including geometric and inertial nonlinearities. Then, Kacem and Hentz [257] reported the experimental observation of a four-bifurcation-point behavior of electrostatically actuated micromechanical resonators. The bifurcation topology tuning can be helpful for any application requiring adjustable stable branches, frequency, bandwidth, or dynamic range. Moreover, Kacem *et al*. [259] investigated the dynamic range enhancement of nonlinear nanomechanical resonant cantilevers for highly sensitive NEMS gas/mass sensor applications. Both geometric nonlinearities and nonlinear electrostatic terms up to the fifth order were modeled to enable the capture of very specific mixed behavior. It provides a novel strategy for NEMS designers that can be used for the enhancement of resonant sensors performances based on the compensation of nonlinearities. In order to compensate for the loss of performance when scaling resonant sensors down to NEMS, it is a challenge to achieve large-amplitude motion of nanomechanical resonators without deteriorating their frequency stability [32,260]. The simultaneous resonances (primary and superharmonic) was used to overcome this limitation, by stabilizing the dynamic behavior of the resonator, which displays reduced dynamic ranges or signal to noise ratio [261]. Uranga *et al*. [263] presented the study and characterization of the non-linear regime of two CMOS-NEMS flexural resonators electrically transduced for mechanical memory applications. More recently, Kacem *et al*. [262] reported experimentally how the combination of the nonlinearity cancellation and simultaneous resonances can be used to stabilize and linearly drive a nanomechanical resonator up to very large amplitudes compared to pull-in limit. This technique may provide one new way towards resonators with high frequency stability for high-performance sensing or time reference.

## 6. Active Frequency Tuning Method

Although substantial development and progress in the transduction techniques [264], an efficient, integrated, and customizable strategy for frequency tuning of micro- and nanomechanical resonators has remained elusive [42]. In order to achieve resonance in the mechanical structure of a MEMS/NEMS resonator, the device must be actuated by an actuator and set to resonate by varying the excitation frequency. Efficient actuation is crucial to obtaining optimal performance. A variety of tuning techniques, including the electrothermal, electrostatic, and magnetic, piezoelectric, optothermal and dielectric excitation methods, have been developed for actuating resonance. The general excitation mechanisms can be divided into local on-chip schemes [51] and approaches depending on external excitations [34]. The former arises from the voltage-induced forces through different types of sources such as electrothermal [265], capacitive, magnetomotive [41], internal piezoelectrical [42,43], or static dipole-based dielectric [44]. The latter implement external excitation such as photothermal [45] or inertia-based piezo-actuated schemes [42].

Table 7 provides the comparison of several actuation mechanisms which are widely used for frequency tuning of resonators [115]. We review these different active mechanisms as well as nonlinear mode coupling that have been developed for frequency tuning of the micro- and nanomechanical resonators.

**Table 7:** Comparison of several actuation mechanisms applied for micro- and nanomechanical resonators.

Actuation Type	Fabrication Process	Power Consumption	Applied Voltage	Current	Nonlinear Effect
Electrothermal	Simple	High	Low	High	Medium
Electrostatic	Simple	Low	High	Low	High
Piezoelectric	Complex	Low	Medium	Low	High
Magnetomotive	Simple	Medium	Low	Medium	High
Dielectric	Medium	High	High	-	High
Photothermal	Complex	Medium	-	-	High

### 6.1. Electrothermal Tuning Mechanism

The resonant frequency of a resonator can be shifted by inducing an expansion or contraction of the structure electro-thermally [85]. The advantages of electro-thermal actuation include simplified fabrication and relatively low operating voltages [266]. Several electrothermal tuning techniques were developed for frequency tuning of microresonators such as resistive heating to introduce thermal strains [19] and localized thermal induced stressing [14]. However, these techniques generally suffer from high power dissipation.

When the electrical current is applied to the resonator, it causes resistive heating and the electro-thermal model can be given by [267]:

(58) $$\nabla (\kappa_{T}A_{t}\nabla T)-\gamma_{T}(T-T_{0})+P^{\prime }=0$$

where $\kappa_{T}$ is the thermal conductivity, $\gamma_{T}$ denotes the net heat loss rate to the substrate per unit length, $A_{t}$ is the cross-sectional area, $T_{0}$ is the room temperature, and $P^{\prime }=I^{2}R_{C}/L$ refers to the Joule heating rate per unit length, in which I is the electrical current, and $R_{C}$ is the resistance.

Both on the relationship of stress and strain, the thermal stress $\sigma_{T}$ for the resonators under Joule heating can be expressed as:

(59) $$\sigma_{T}=-2\int_{0}^{L/2}\alpha ET^{\prime }(x)dx$$

where α is thermal expansion coefficients (TEC) and $T^{\prime }(x)$ is the derivative of temperature. For the case of nanotube resonators, Pop *et al*. [267] deduced the temperature profiles $T(x)=T_{0}+P^{\prime }/\gamma_{T}[1-\cosh(x/L_{H})/\cosh(L/2L_{H})]$, where $L_{H}=\sqrt{\kappa_{T}A_{t}/\gamma_{T}}$ is the characteristic thermal healing length. Mei and Li [18] recently presented the temperature profile a $T(x)=T_{0}+P^{\prime }/(\gamma_{T}+\alpha P^{\prime })[1-\cosh(x/L_{H})/\cosh(L/2L_{H})]$, where $L_{H}=\sqrt{\kappa_{T}A_{t}/(\gamma_{T}+\alpha P^{\prime })}$. If the material has a uniform change ΔT, the change in length $\delta_{L}$ yields $\delta_{L}=\alpha L\Delta T$, where L is the initial length of the material. When the expansion of the material is affected by the interaction to another material, the thermal stress $\sigma_{T}$ can be simplified as $\sigma_{T}=-\alpha E\Delta T$ [85]. Under the effect of thermal stress, the resonant frequency can be given by [16]:

(60) $$f_{\sigma }=f_{0}\sqrt{1+\frac{\sigma L^{2}}{3.4Eb^{2}}}$$

where σ is the tensile stress, and it is the sum of the initial tensile stress $\sigma_{i}$ from the film deposition process and the thermal stress $\sigma_{T}$ from heating of the structure. Jun *et al*. [16] provided a single expression for the thermal tuning of a beam as follows:

(61) $$f_{\sigma }/f_{i}=\sqrt{1+{\left(\frac{f_{0}}{f_{i}}\right)}^{2}\left(\frac{\alpha_{e}}{K_{e}}\frac{L^{3}}{40.8b^{3}h}\right)P_{e}}$$

where $f_{i}$ is the initial measured frequency under the initial tensile stress and $f_{i}=f_{0}\sqrt{1+\sigma_{i}L^{2}/(3.4Eb^{2})}$, $\alpha_{e}$, $K_{e}$ and $P_{e}$ are the effective thermal expansion coefficient, thermal conductivity, and electrical heat production, respectively.

**Figure 19:**
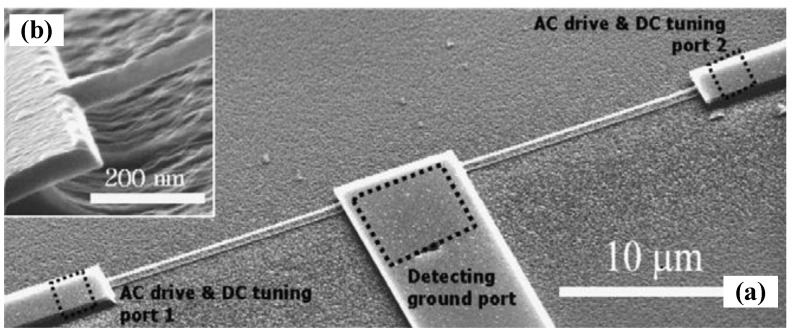
(**a**) SEM of a completed nano-mechanical resonator [16]. The nanobeams with two AC driving and DC tuning ports and a detection port; (**b**) Close-up view of the suspended structure. Reused with permission from [16], Copyright 2006, IOP Publishing.

As shown in Figure 19, Jun *et al*. [268,269] used this effective electrothermal tuning method, which was widely applied to tune frequency of MEMS devices [14,19], for composite nanomechanical resonators with ultrathin 3C-SiC films and 30–195 nm of aluminium [1,27]. A DC tuning voltage up to 100 mV was applied in parallel with the RF drive for the electrothermal tuning [270]. The direct current heats the beam, and thus changes the stress and decreases the resonant frequency, which was tuned by 10% with Joule heating of DC current [268]. Upon heating, the quality factor decreases by up to ~8% [16]. The resonant frequency shifts about 6.5% at 31 kHz using electro-thermal stiffness change [19]. A tuning range of up to 1.1% from the frequency of 39.2 kHz with 54 mW power was reported in [271]. The SNR level decreases almost linearly with the increase of tuning power [269]. In addition, the effect of frequency tuning by magnetic field was controlled by interplay between stress- and shape-induced anisotropy energies [272].

The resonant frequency of the resonator with the thermal axial load $T_{f}$ can be expressed as [270,273]:

(62) $$\omega_{0}=2\pi f_{0}={\left(\frac{2\pi }{L}\right)}^{2}\sqrt{\frac{EI}{3\rho A}\left[1+\frac{T_{f}}{EI}{\left(\frac{L}{2\pi }\right)}^{2}\right]}$$

The tension $T_{f}$ in the resonator beam after the dc heating changes from $T_{h0}$ to:

(63) $$T_{f}=T_{h0}-k_{h}EAV_{dc}^{2}$$

where $k_{h}={\left(L/\pi \right)}^{2}/(mC_{p}R)$, in which $C_{p}$ is the specific heat conductivity, m is the mass of the resonator, and R is the resistance. It notes that electrothermal heating is one of radio frequency tuning method in nanomechanical resonators with magnetomotive transduction and can modify the dynamic range of the resonators [273,274,275,276].

**Figure 20:**
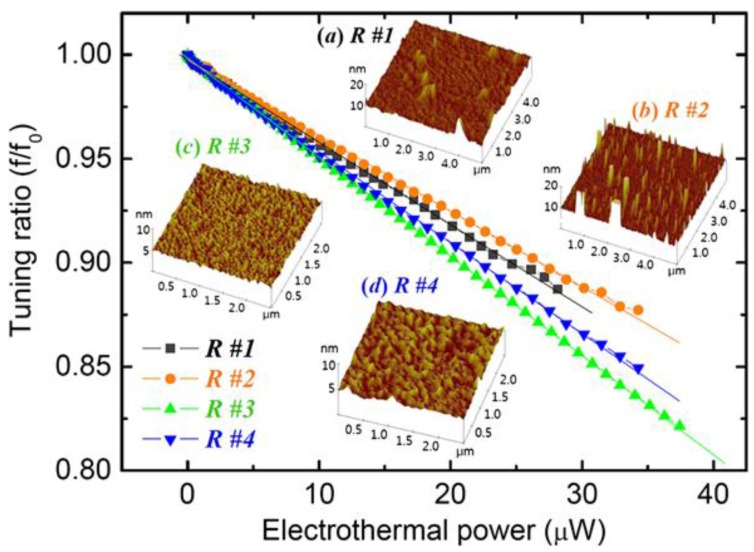
Frequency tuning performance as a function of surface roughness of the nanobeam resonator [275]. The average roughness of the samples (*a*) R#1: 11.2nm, (*b*) R#2: 28.8 nm, (*c*) R#3: 0.9nm, and (*d*) R#4: 2.4nm, observed in AFM image of surface morphology. Reused with permission from [275].

As illustrated in Figure 20, tuning power and surface roughness have a significant role on the frequency tuning properties. On one hand, when the tuning power was supplied, the resonant frequency of the resonator was tuned downward due to the Joule heating [275]. On the other hand, the surface roughness is an important parameter influencing the resonant frequency and tuning performance. The surface roughness makes the loss of resonating performance more complicated to predict. It demonstrated that the dissipation prevails more on a rougher surface due to the effects of electron scattering, energy loss, and unequal or non-uniform electrothermal heating [270,275].

Localized heating [277,278] was demonstrated to be one effective method for frequency tuning of micromechanical resonators. The possibility of programming multiple eigenfrequency states of the microresonator with localized Joule effect was also reported [279]. More recently, frequency self-tuning of CNT resonator using joule heating mechanism was demonstrated to improve the mass detection sensitivity from 1.783 MHz/zg to 5.013 MHz/zg [18].

### 6.2. Electrostatic Tuning Mechanism

#### 6.2.1. General Tuning Model: Electrode Geometry

Electrostatic actuation remains an attractive method for frequency tuning due to virtually nonexistent current loss, high energy density, and large force at micro-scale [280]. Since the pioneering experiments with charged soap-bubbles reported by Taylor [281] and the invention of the first microresonators [282], electrode geometries and support configurations in resonators have developed significantly [283]. Electrode geometry has changed from being planar (graphene electrodes [148]), to cylindrical (carbon nanotubes (CNT) [214,284] and nanowires [17,32]), to an array of cylinders (nanowire arrays [285], nanotube arrays [286]), and to fractal [283,287].

**Figure 21:**
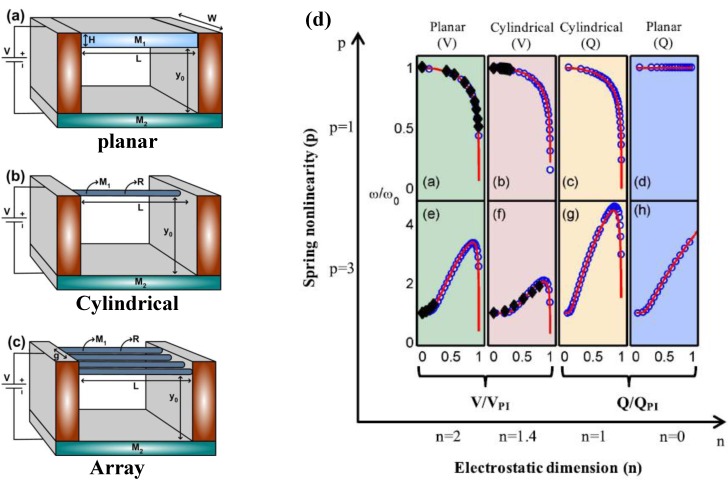
General electrode geometries for micro- and nano-mechanical resonators reported by Jain and Alam [288]. (**a**) Classical planar electrode; (**b**) Cylindrical electrode; (**c**) array of cylinders; (**d**) Resonant frequency as a function of (V/Q) with scaling effect. Reused with permission from [288].

The resonant frequency can be tuned by the applied voltage and its dependence is well understood within the continuum mechanics framework [286]. Palit *et al*. [283] and Jain and Alam [288] presented a general framework to analyze electromechanical actuators having arbitrary electrode geometries and support configurations and reported the universal scaling relationships for resonant frequency depending on the scaling parameters. Figure 21a–c show the schematic of various movable electrodes $M_{1}$ is suspended above a fixed bottom electrode $M_{2}$. The governing equation for the deflection y of $M_{1}$ with Young’s modulus E, Poisson’s ratio ν, thickness H, and subjected to an externally applied voltage V is given by [283]:

(64) $$\frac{EH^{3}}{12(1-\nu^{2})}\nabla_{r}^{4}y=-\frac{1}{2}\frac{dC(r,y)}{dz}V^{2}$$

where r is a vector in the plane of $M_{1}$ and C(r,y) is the capacitance per unit area between $M_{1}$ and $M_{2}$ at position r and given by $C(r,y)=\varepsilon_{0}\beta_{e}L^{n-2}/{\left(y+T_{d}\right)}^{n-1}$, in which n is the electrostatic dimension parameter that defines the electrostatics of the system, $T_{d}$ is the effective dielectric thickness normalized by the dielectric constant, $\beta_{e}$ is a constant that depends exclusively on the geometrical configuration of the electrodes.

With the increase of applied voltage V, separation between the two electrodes (y) is dictated by the balance of restoring spring and electrostatic forces. The application of potential ϕ V or Q) not only changes the separation, but also modifies the effective stiffness $K_{eff}=-(d/dy)(F_{S}-F_{elec})$. The tuning of the resonant frequency with scaling effect can be given by [288]:

(65) $$f_{n}=f_{0}{\left[\sum_{p=1}^{p=\infty }\frac{k_{p}}{k_{1}}{\left(y_{0}-y\right)}^{p-1}\left(p+n-n\frac{y_{0}}{y}\right)\right]}^{1/2}$$

where $y_{0}$ is the initial air-gap $k_{1}$ is the linear spring stiffness, $k_{p}$ is the spring constant associated with the nonlinearity of order p. Figure 21d illustrates the scaling relationship between the resonant frequency and applied potential V (or Q) for various electrode geometries (planar and cylindrical electrodes). The expression Equation (65) reduces to $f_{n}=f_{0}{\left[3-(2y_{0}-y)\right]}^{1/2}$ for a well-known resonant gate transistors [282] with linear spring n=2 and $k_{p}\approx 0$) [288]. Therefore, the resonant frequency of the resonators can electrically be tuned by applying a voltage between the electrodes.

#### 6.2.2. Single-Electron Tuning

With the fields of electronics and mechanics making impressive progress toward quantum mechanical devices [289], the combination of electronic transport with mechanics has become one of the exciting new areas during the past several years [290]. The resonant frequency can be tuned by using an external electric means, which is convenient for practical applications [291,292].

**Figure 22:**
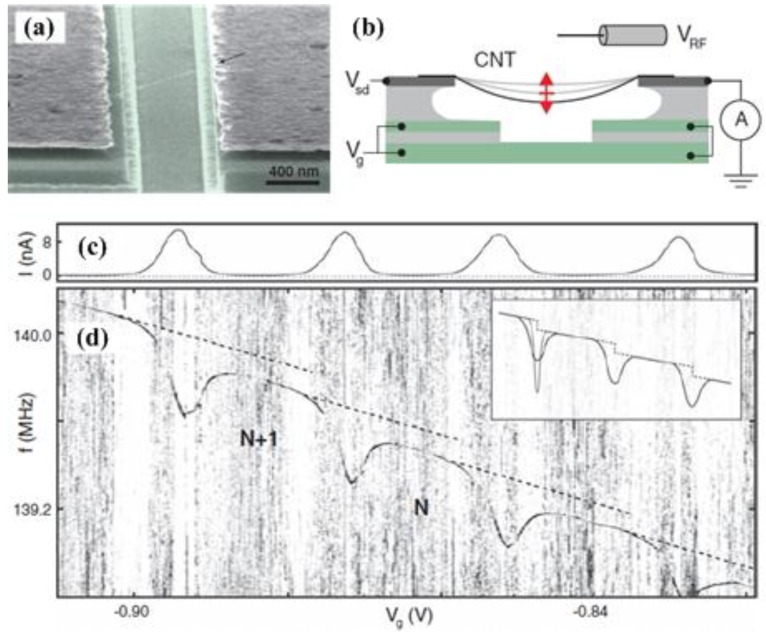
Single-electron tuning mechanism developed by Steele *et al*. [293]. (**a**) Nanotube mechanical resonator; (**b**) Device geometry for single-electron tuning; (**c**) Single-electron Coulomb blockade oscillations; (**d**) Tuning the resonant frequency with gate voltage. Reused with permission from [293], Copyright 2009 American Association for the Advancement of Science.

Single-electron charge fluctuations can create periodic modulations of the resonant frequency of mechanical resonators [293]. Steele *et al*. [293] and Lassagne *et al*. [291] have found that the resonant frequency and dissipation in the nanotube mechanical resonators are both highly sensitive to the charge state with single electrons. As shown in Figure 22, as the voltage is applied to the gate, the electrostatic force acting on the nanotube can be expressed as [294]:

(66) $$F_{elec}=\frac{1}{2}\frac{dC_{g}}{dx}{\left(V_{g}-V_{g,AC}\right)}^{2}=\frac{1}{2}\frac{dC_{g}}{dx}\left(V_{g}^{2}+\frac{1}{2}V_{g,AC}^{2}+2V_{g}V_{g,AC}\cos(2\pi \omega_{e}t)+\frac{1}{2}V_{g,AC}^{2}\cos(4\pi \omega_{e}t)\right)$$

where $C_{g}$ is the capacitance between the nanotube and the gate, x is the displacement of the fundamental model of the nanotube, and $V_{g}$ and $V_{g,AC}$ (with frequency $\omega_{e}$) are the applied voltages on the gate and the nanotube, respectively. The resonant frequencies increase with gate voltage owing to the tensioning of the resonator [31] and is tuned by more than a factor of 2 with the gate voltage [293]. In the limiting cases, the fundamental resonant frequency can be given by [295]:

(67) $$\omega_{n}=\sqrt{\frac{EI}{\rho A}}\{\begin{array}{cc} 22.38L^{-2}+0.28\xi^{2}, & \xi L\ll 1\\ \pi \xi L^{-1}+2\pi L^{-2}, & \xi L\gg 1 \end{array}$$

where the variable $\xi =\sqrt{T/EI}$. The resonant frequency dependence $\omega_{n}\propto L^{-2}$ is associated with a loose string, while $\omega_{n}\propto L^{-1}$ represents that the string is tied.

When a single electron is added to the suspended CNT, the resonant frequency of the resonator dips dramatically due to the coupling between electronic and mechanical motion, which causes a softening of the spring constant. However, the resonant frequency changes non-smoothly and shows discrete jumps (Figure 22d) due to the Coulomb blockade [289]. This is the first experimental observation the electromechanical coupling of an electron in the quantum mechanical regime via CNT-based NEMS device. Solanki *et al*. [103] observed the nonmonotonic dispersion of the resonant frequency with dc gate voltage in nanowire resonators. More recently, Benyamini *et al*. [296] explored a new generation of suspended carbon nanotube mechanical resonators with wide-ranging local control. The key parameter in these experiments is the possibility of tuning the resonant frequencies by external gate electrodes [214,289,290,291,293]. These gate electrodes can not only cause the microtubes to vibrate at their resonance modes, bust also introduce an axial strain which modifies the resonant frequencies significantly [295,297].

#### 6.2.3. Capacitive Softening Effect

**Figure 23:**
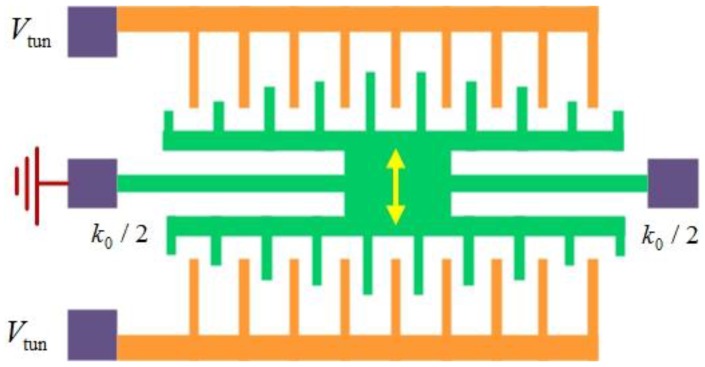
Resonant frequency tuning by capacitive softening effect.

Resonant frequency of parallel-plate capacitive resonators can be tuned by changing the DC voltage applied across the sense and drive capacitive gaps. The principle is based on electrostatic force acting on the mass [298], as shown in Figure 23. The resonator structure contains an extra electrode system for resonant frequency tuning by applying a DC voltage and the tuning voltage $V_{tun}$. The total stiffness $k_{total}$ of the tuned resonator can be calculated as:

(68) $$k_{total}=k_{0}-\frac{1}{2}\frac{d^{2}C(x)}{dx^{2}}V_{tun}^{2}$$

where $k_{0}$ is the mechanical stiffness and C(x) is the total capacitance between the two electrodes. The forces lead to a softening of the resonator system and result in decreasing the resonant frequency. A maximum tuning voltage of 35 V is required for continuous resonant frequency tuning from 1 to 10 kHz and a maximum resonance frequency shift of 0.7% [298]. With sub-100 nm self-aligned vertical capacitive gaps designed for the first time, the resonant frequency can be tuned from 505 kHz to 450 kHz for the in-plane resonators by changing the DC voltage and providing a large electrostatic tuning range about 10% [299]. The resonant frequency can be tuned down to −75% of its maximum value using electrostatic softening effect [300]. Several measurements on the suspended metallized SiC beam, clamped-clamped InAs nanowire resonators [15,103], and CNT mechanical resonators [155] have also displayed the decrease of resonant frequency due to the electrostatic softening of the vibration.

Recently, Eriksson *et al*. [301] discussed the coupled effects of deflection-induced tension and electrostatic softening on the resonant frequency tuning of circular nanomechanical graphene resonators.

#### 6.2.4. Combination of Hardening and Softening Effects

Micro- and nano-resonators actuated by electrostatic force can offer *in situ* frequency tuning over wide frequency range [31,108,184]. The frequency tuning is known to be governed by two distinct mechanisms: the elastic hardening effect, which arises in the presence of large elastic deformations and increases the resonance frequencies, and the capacitive softening effect, which is inherent to the electrostatic actuation force and decreases the resonance frequencies [15,302,303]. The resonant frequency has been observed to tune either upward [31] or downward [10], and both states [15,215].

**Figure 24:**
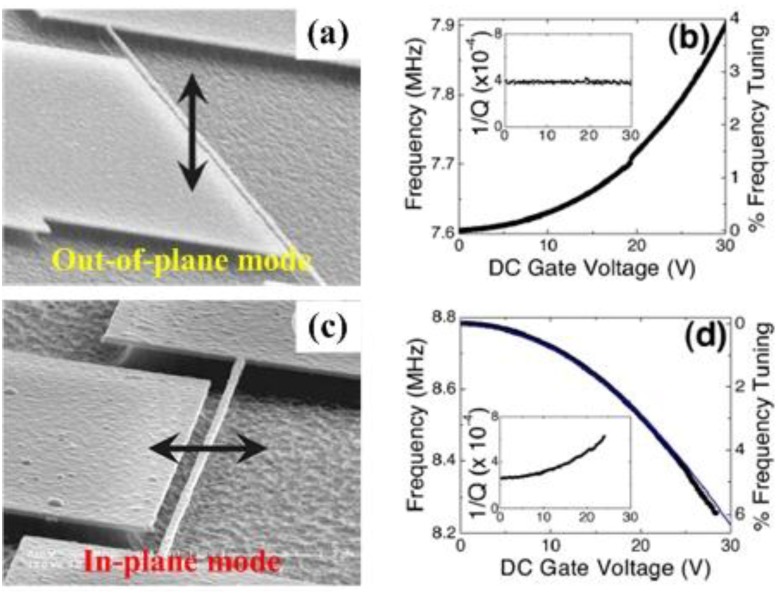
Electrostatic tuning setup and measurement reported by Kozinsky *et al*. [15]. (**a**) The beam’s vibration out-of-plane with the gate; (**b**) Elastic tuning of resonant frequency upward; (**c**) The beam’s vibration in-plane of the gate; (**d**) Capacitive tuning of resonant frequency downward. Reused with permission from [15], Copyright 2006, American Institute of Physics.

As one of the most representative electrostatic tuning methods, Kozinsky *et al.* [15] demonstrated an ability to tune the resonant frequency of resonators both upward and downward. Figure 24 illustrates the electrostatic tuning setup and measurement, and shows the results obtained by varying the DC bias applied to the gate electrode. Both softening and hardening types of frequency tuning in the resonator were observed. As shown in Figure 24a,b, the resonant frequency of the beam with gate voltage increases when the out-of-plane mode of vibration occurs. As the applied DC gate voltage increases, the resonant frequency increases. For the in-plane mode, it can be observed from Figure 24c,d that the decrease in the resonant frequency. The clamped-clamped beam is electrostatically attracted to the gate and results in the spring constant becoming smaller. Under the combination effect of electrostatic force and elastic restoring force, the governing equation can be given by [15]:

(69) $$EI\frac{\partial^{4}w(x,t)}{\partial x^{4}}+\rho A\frac{\partial^{2}w(x,t)}{\partial t^{2}}-\left[T_{0}+T\left(\frac{\partial w(x,t)}{\partial x}\right)\right]\frac{\partial^{2}w(x,t)}{\partial x^{2}}=F_{ext}(x,t)$$

where T is the residual tension, T(∂w(x,t)/∂x) is the bending-induced tension and $T(\partial w(x,t)/\partial x)=(EA/2L)\int_{0}^{L}{\left(\partial w(x,t)/\partial x\right)}^{2}dx$, the electrostatic force $F_{ext}(x,t)=c_{z}[z(x,t)]V^{2}/2$, in which c[z(x,t)] is the capacitance per unit length and z(x,t) is a time-varying AC displacement. Electrostatic force on the nanotube leads to a deflection towards the gate and results in the increased mechanical tension [31,294]. The resonant frequency can be obtained as [15]:

(70) $$\omega_{n}=2\pi f_{n}={\left(\frac{2\pi }{L}\right)}^{4}\left(\frac{EI}{3\rho A}+\frac{EA_{dc}^{2}}{6\rho }\right)+\frac{T_{0}}{3\rho A}{\left(\frac{2\pi }{L}\right)}^{2}-\frac{K_{C}V^{2}}{\rho A}$$

where $K_{C}$ is the capacitance expansion coefficient, $A_{dc}$ is static deflection amplitude. The different frequency tuning behaviors can also explained by Equation (70). The frequency increases in the out-of-plane mode due to increasing the gate voltage, which only stretches the beam. For the in-plane vibration mode, both stretching and electrostatic attraction occurs. Electrostatic attraction to the gate has the softening effect on the resonator for lower gate voltage before the hardening due to the stretching effect [15]. The theoretical expression for resonant frequency Equation (70) agrees well with the experimental data as shown in Figure 24d. The limitation of frequency tuning using electrostatic spring softening effect is that only one directional tuning is available.

On one hand, when a DC bias voltage $V_{dc}$ is applied to the gate and a potential difference is created, the resulting electrostatic force attracts the resonator toward the gate and results in induced-tension in the resonator. The elastic hardening effect increases the resonant frequency which can be given by [215,302]:

(71) $$f_{n}={\left({f_{0}}^{2}+\frac{\pi^{2}EZ_{dc}^{2}}{4\rho L^{4}}\right)}^{1/2}=f_{0}+A_{e}V_{dc}^{2}$$

where $Z_{dc}$ is the static displacement of the center of the beam and $Z_{dc}=\left[\frac{1}{2}C^{\prime }LV_{dc}^{2}\right]/(32Ed_{w}^{4}/L^{3})$, in which $C^{\prime }$ is the first derivative of capacitance, $d_{w}$ is the diameter of the cylindrical wire.

On the other hand, when the DC voltage $V_{dc}$ is applied to side gate, the electrostatic force is in the direction of the resonator and the bias not only creates tension but also results in the capacitive softening effects [302]. The capacitive coupling between the electrodes allows electrostatic control of both the mean position of the resonator and its resonance frequency. The frequency dependence of the capacitive softening effect can be expressed as [15,215,302]:

(72) $$f_{n}={\left({f_{0}}^{2}-\frac{C^{\prime \prime }V_{dc}^{2}}{8\pi^{2}\mu_{m}L}\right)}^{1/2}={\left({f_{0}}^{2}-B_{c}V_{dc}^{2}\right)}^{1/2}$$

where $C^{\prime \prime }$ is the second derivative of capacitance.

**Figure 25:**
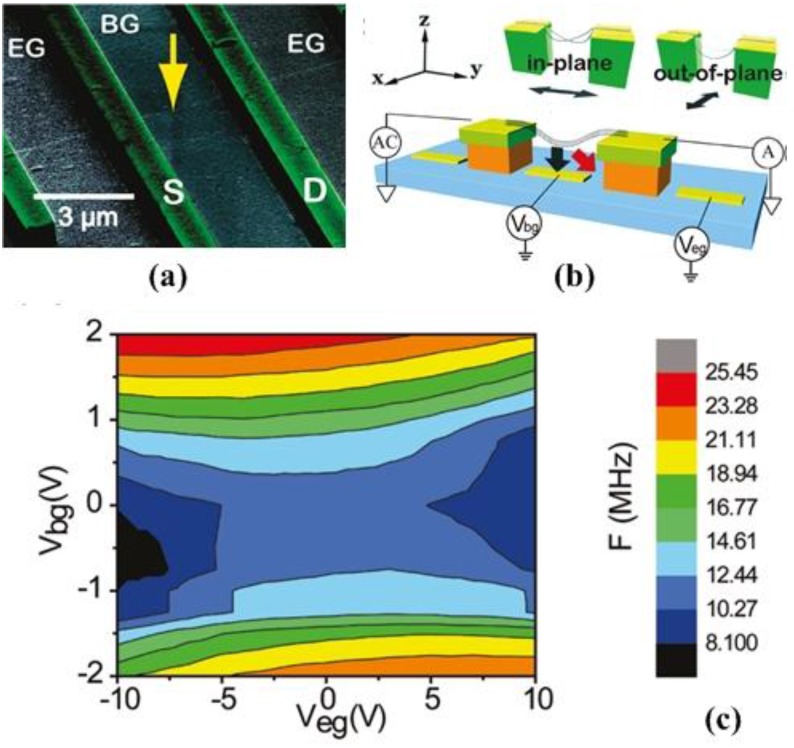
Device geometry and dual-gate frequency tuning of single-walled carbon nanotube (SWNT) resonator reported by Wu and Zhong [215]. (**a**) Dual-gate nanotube resonators; (**b**) Frequency tuning through both elastic hardening and capacitive softening mechanisms; (**c**) Resonant frequency as a function of $V_{eg}$ and $V_{bg}$. Reused with permission from [215], Copyright 2011, American Chemical Society.

SWNT resonators are known to have multiple vibrational states, including out-of-plane, in-plane, and the higher order modes [31,215]. Wu and Zhong [215] first investigated the frequency tuning using bottom-gate electrode, and reported the observation of the dual-gate nanotube resonators (Figure 25a) can realize frequency tuning through both elastic hardening and capacitive softening mechanisms, as illustrated in Figure 25b. The coupling of bottom-gate (BG) and end-gate (EG) effects on the capacitive softening was taken into account. The capacitive softening equation can be modified by including the effect of elastic hardening and an offset voltage $V_{0}$ as:

(73) $$f_{n}={\left[{\left(f_{0}+A_{e}V_{bg}^{2}\right)}^{2}-B_{c}{\left(V_{eg}-V_{0}\right)}^{2}\right]}^{1/2}$$

where $A_{e}$ and $B_{c}$ are the elastic hardening tuning and capacitive softening coefficients, respectively. Figure 25c shows a two-dimensional plot of resonant frequency as a function of $V_{eg}$ and $V_{bg}$. The resonant frequencies illustrate symmetric tuning around gate voltages corresponding to the charge neutral point.

#### 6.2.5. Frequency Tuning for Comb-Drive Microresonators

To achieve wide frequency tuning range with low power consumption, many researchers have focused on various comb finger designs. Various frequency tuning methods have been applied and demonstrated for comb-drive microresonators, as listed in Table 8.

Electrostatic comb structures have been the key design elements for comb-drive micromechanical resonators [304,305,306,307,308,309,310,311,312,313,314,315]. Multiple tuning approaches have been presented to tune the effective spring constant of the resonators, including parallel-plate [20], fringing field [304], and variable gap comb-drives [305,306]. Lee and Cho [21] applied a control voltage for triangular electrostatic comb arrays for 3.3% reduction in the resonant frequency. Adding material deposition to the mass can lead to 1.2% resonant frequency reduction [89]. The use of constrained thermal expansion can result in 50% increase in the resonant frequency under vacuum and 25% frequency reduction at atmospheric pressure [19]. These tuning methods provide small change in resonant frequency [21,89] or require high power for frequency tuning operations [19]. Furthermore, under the applied external electrical potential and Joule heating effects, the resonant frequencies of the comb drive resonators change from 22.2 kHz to 16.2 kHz, resulting in the 27% reduction in the resonant frequency [307].

**Table 8:** Various frequency tuning methods for the comb-drive microresonators [19,21,89,298,305,310,311,313,314,315].

Reference	Tuning Mechanism/Method	Geometry Configuration	Active/Passive Method	Resonant Frequency	Tuning Range
Lee and Cho [21]	The DC-biased electrostatic tuning comb structures arranged in the triangular shape to adjust the resonant frequency using the linear electrostatic force.	Triangular comb arrays	Active method	2.42 kHz	−3.3% (measurement); −5.3% (estimation)
Syms [19]	The use of constrained thermal expansion to tune the resonant frequency. The tensile strains may be set up in the suspension using a folded geometry.	Folded geometry	Active method	1.56 kHz	−25% (at atmospheric pressure); 50% (at 10 mTorr)
Lee *et al.* [313]	A closed-form design approach for comb finger profiles to achieve constant electrostatic stiffness or linear electrostatic force.	Curved comb finger	Active method	19 kHz	−55% (measurement); −45.4% (theoretical result); −42.7% (simulation)
Jensen *et al.* [305]	Shaped fingers allow the design of resonators operating at a wide range of spring stiffness and tuning resonant frequency over a large range.	weakening fingers	Active method	4.3 kHz	165 Hz (downward)
		stiffening fingers			5.3 kHz (upward)
Xu and Tsai [310]	The basic idea is to synthesize the design of the supported springs and the releasing holes in the proof mass. Then the process-induced effective spring constant variation can be balanced by effective mass variation.	DRIE-induced variation	Active method	3.28 kHz	2.1%
Zine-El-Abidine and Yang [311]	The suspension configuration can be mechanically altered to change its spring constant.	Curved electrode	Active method	10.8 kHz	17.6 kHz (two actuators); 21.4 kHz (four actuators)
Scheibner *et al.* [298]	The principle is based on electrostatically generated, amplitude-dependent forces acting on the seismic mass, and the structures implement electrostatic softening effect.	Capacitive surfaces	Active method	2.89 kHz	901 Hz
Morgan and Ghodssi [314]	The vertically-shaped comb-fingers were designed as electrostatic springs without increasing the device area.	Shaped fingers	Active method	1.6 kHz	17% (bidirectional)
Joachim and Lin [315]	The selective deposition of polysilicon by silane decomposition on electrically heated, released microstructures.	Selectively deposited polysilicon	Passive method	86.6 kHz	1.96%
Chiao and Lin [89]	Post-packaging tuning process for microresonators by pulsed laser deposition (PLD). The advantages include precise process control, versatility and easy implementation.	donor film structure	Passive method	12.37 kHz	−1.2%

Various comb finger configurations had been designed to achieve wide frequency tuning with low power consumption [316]. The shaped comb design was previously made to maximize the electrostatic force by a segmented comb with different widths [306]. Adams *et al*. [39] presented four electrostatic actuators to change the stiffness and tune the resonant frequency upwards to 146% and downwards to 7.7% of the original values. The planar shaped combs for delivering electrostatic force with specific nonlinearity and the resonant frequency can be tuned either downwards or upwards [305]. A specific varying-gap comb-finger design for a large-stroke parametric resonator was recently reported in [308]. A new truss electrode presented by Khirallah [309] can produce time-varying electrostatic axial force on the resonator and consequently modulate its effective spring constant. Xu and Tsai [310] presented an interesting method to seek the proper parameter sets to balance the variations of process-induced spring constant and mass. The resonant frequencies are in the range of 32 102 ± 25 Hz and obey basically the normal distribution. The stiffening or the weakening of the comb allows the resonant frequency to be swept between the multiple frequencies of the comb achieving a very wide tuning range [311]. Zhong *et al*. [312] demonstrated an interesting result of the inclination effect on the frequency tuning of comb-driven resonators. Although these methods obtained tunability, small variation of the resonant frequency can be tuned for the micromechanical resonators.

In addition, the ultimate limit to electrostatic tuning in micro- and nano-resonators depends on the pull-in effect when the beam structure gets close enough to the gate [15]. The major drawback of this scheme is that the response is nonlinear. On the contrary, electrostatic pull-in can also be used for micro- and nanomechanical resonators [316]. Kafumbe *et al*. [40] explored a new method of actively tuning the resonant frequency of microresonators using electrostatic pull-in to adjust the length of the resonating structure and guarantees frequency tuning throughout the lifetime of the device. Ke [317] presented the theoretical investigation of double-sided excitation scheme for resonant frequency tuning of nanotube resonators with table range reaching up to 90% of the gap between the actuation electrodes, which exceeds the resonant pull-in limit. Pull-in has also been proposed for sensing adsorbate stiffness in nanomechanical resonators [318].

#### 6.2.6. Photothermal Tuning Mechanism

Optical techniques have the advantages of requiring no electrical connections, possessing the highest resolution, and can be implemented for measurements of the resonators in vacuum, gas, and liquids [23,319]. The resonant properties of micro- and nano-mechanical resonators driven by the photothermal approach contain rich information about mechanical and thermal properties of the resonating system [320,321]. The resonant frequencies of nanocantilevers can be influenced by the optical pressure and photothermal force generated by the laser beam probe [94,322,323].

The photothermal effect can be used to tune the frequency of micro- and nano-resonators [45,324,325,326]. Kim *et al*. [325] demonstrated the pressure-sensing scheme based on the photothermal effect in the miniaturized beam resonator, and observed the considerable decrease in the resonant frequency due to the photothermally induced compressive stress. Incident optical power results in the temperature rise in the composite beam and the shift in the resonance frequency due to thermal stress [324]. Photothermal actuation was also used for the self-excitation and for measuring the resonant frequency of nanomechanical resonators in liquids [94,327]. The immense resonant frequency tunability available by this technique may be of importance for numerous NEMS applications [45].

To understand the effect of the laser power on the dynamic response of the resonators, the resonance spectra at multiple harmonic modes were taken at various power levels [324]. Kim *et al*. [324] determined the amount of incident power $P_{\text{laser}}$ on the beam by considering the Gaussian distribution as:

(74) $$P_{\text{laser}}=(1-R_{r})\int_{-b/2}^{b/2}\int_{L/2}^{L/2}I_{m}\exp\left[-\frac{2(x^{2}+y^{2})}{r_{o}^{2}}\right]dxdy$$

where $R_{r}$ is the reflectivity, $I_{m}$ is the maximum intensity from the total optical power measurement, $r_{o}$ is the experimental optical spot size. The corresponding temperature distribution T(x,t) caused by the laser power can be calculated from the heat conduction model with Gaussian thermal source as:

(75) $$\frac{\partial T(x,t)}{\partial t}=\frac{c_{t}}{\rho_{p}c_{p}}\frac{\partial^{2}T(x,t)}{\partial t}+\frac{P_{\text{laser}}}{\rho_{p}c_{p}Lbd_{t}}-\frac{2(b+d_{t})c_{h}}{\rho_{p}c_{p}bd_{t}}\left[T(x,t)-T_{0}\right]$$

where $c_{t}$, $\rho_{p}$ and $c_{p}$ are the weighted average of thermal conductivity, mass density and heat capacity, respectively, $d_{t}$ is the total thickness, $T_{0}$ is the ambient temperature, and $c_{h}$ is the heat transfer coefficient. The observed resonant frequency decreases with the optical power due to the heating of the resonator, generating the photothermal stress. Under the axial stress, the resonant frequency of a clamped-clamped beam at nth mode can be given by [324]:

(76) $$f_{n}=\frac{{\left(2n+1\right)}^{2}\pi }{16\sqrt{3}}\frac{d}{L^{2}}\sqrt{\frac{E}{\rho }}\sqrt{1+\frac{11.6}{{\left(n+1\right)}^{2}\pi^{2}}\frac{L^{2}}{Ed^{2}}\sigma }$$

where the axial stress σ is a sum of the intrinsic stress $\sigma_{i}$ due to the SiN layer deposition and fabrication processes, and the temperature-dependent thermal stress $\sigma_{th}$. The effect of the photothermal stress on the dynamics of the beam resonator can be more easily found in the square of the resonant frequency ${f_{n}}^{2}$. It can be clearly observed that the linear dependence of ${f_{n}}^{2}$ on ΔT at several modes, which is expected from Equation (76). The inset shows the average temperature rise ΔT as a function of incident optical power $P_{\text{laser}}$ calculated from the heat conduction model with a Gaussian thermal source.

**Figure 26:**
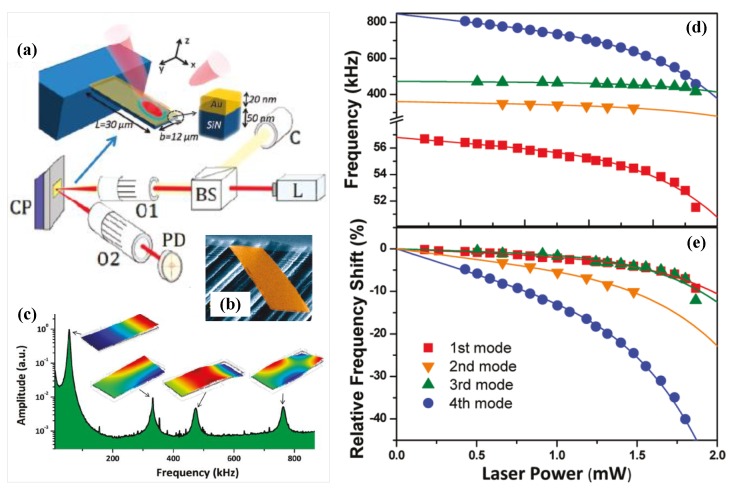
Laser-induced resonant frequency shift reported by Pini *et al*. [319]. (**a**) Schematic diagram of the structure and dimensions of the laser beam deflection technique; (**b**) SEM of the fabricated bimetallic cantilever; (**c**) Frequency spectrum of the thermomechanical fluctuations; (**d**) First four resonant frequencies of the cantilever as a function of the laser power intensity; (**e**) The relative frequency shifts. Reused with permission from [319], Copyright 2011, American Chemical Society.

Notably, Pini *et al*. [319] indicated that the light back-action effect is very significant in ultrathin bimetallic cantilevers and demonstrated that the laser beam used for probing the mechanical state of nanomechanical resonators can extraordinarily shift the resonant frequencies. Figure 26a illustrated the schematic of the optical detection and the cantilever structure (Figure 26b). A typical frequency spectrum of the thermomechanical noise of the fabricated cantilevers was shown in Figure 26c. Figure 26d,e plotted the first four resonant frequencies and frequency shift of the cantilever as a function of the laser power. It can be found that the laser power increase gives rise to a decrease of the resonant frequency of about 30%. Interestingly, the resonant frequency shifts follow a nonlinear behavior with the laser power. The tunable optical gradient force can also cause the frequency shift in resonators [328]. In addition, the frequency shift due to the laser back-action effect follows a nonlinear behavior, which reveals a new mechanism of resonance frequency shift due to in-plane stress.

#### 6.2.7. Piezoelectric Tuning Mechanism

As one of the earliest and most straightforward actuation methods, piezoelectric transduction technique provides a means of directly converting an electric field into mechanical strain [42]. The piezoelectric transduction technique and actuation scheme also enables the resonant frequency of the mechanical oscillator to be tuned [329,330]. The ability to control the effective spring constant using the piezoelectric effect enables excitation of the fundamental mode through parametric resonance [43,331].

**Figure 27:**
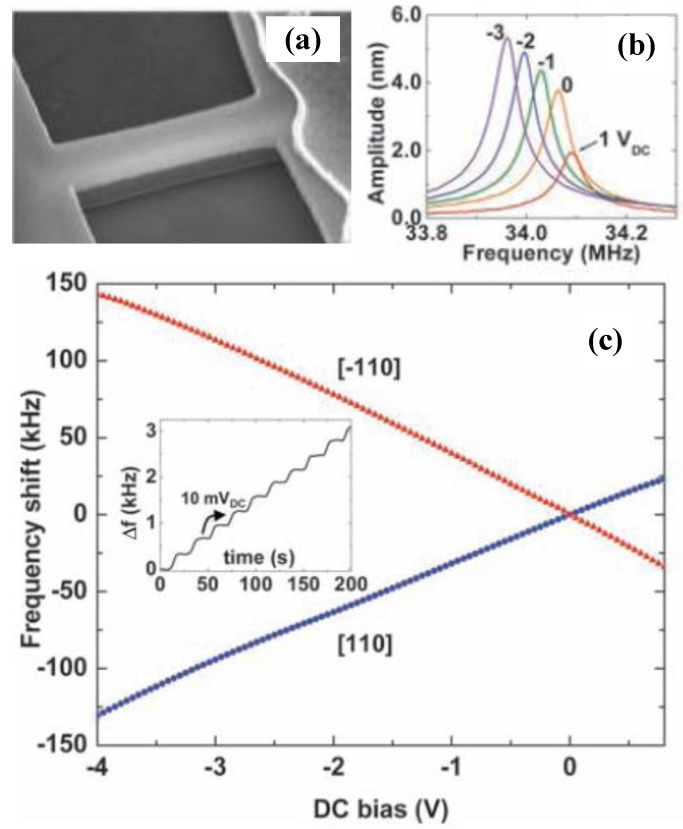
Piezoelectric resonant frequency control and tuning [42]. (**a**) Clamped-clamped beam which used to gauge the efficiency of piezoelectric excitation; (**b**) Frequency response near the fundamental out-of-plane resonant mode; (**c**) Measurements of resonant frequency shift as a function of DC bias voltage. Reused with permission from [42], Copyright 2007 American Association for the Advancement of Science.

Using a p-type/intrinsic/n-type diode structure, Masmanidis *et al*. [42] investigated the use of piezoelectric semiconductors as active structural materials for nanomechanical resonators. The remarkable feature due to the piezoelectric effect is voltage-induced resonant frequency control, as shown in Figure 27. Resonant frequency shifts are clearly observed upon the DC-biasing of the clamped-clamped beam resonator (Figure 27b). In the case of small perturbations, frequency shift $\Delta f_{piezo}$ can be quantitatively expressed as:

(77) $$\Delta f_{piezo}=-\frac{d_{3j}V}{2\pi t_{p}}\sqrt{\frac{3E_{p}}{\rho }}$$

where $d_{3j}$ is the anisotropic piezoelectric coefficient, $E_{p}$ is the elastic Young’s modulus and ρ is the density $t_{p}$ is the total device thickness, V is the DC bias voltage. It can be found that this expression implies linear frequency-voltage dependence.

Due to the anisotropic nature of the piezoelectric coefficient, the slop of Δf can be controlled and tuned by fabricating the beam along a prescribed direction [42], as illustrated in Figure 27c. The equal and opposite tuning slope of devices aligned along the (110) and (–110) directions is characterized by the opposite sign of $d_{3j}$ along these directions. In addition, the potential application of voltage-dependent frequency tuning for piezoelectric nanomechanical charge sensing was also demonstrated. However, the bandwidth of a piezoelectric actuator depends upon the relaxation time of the deformations after the electric field is removed [264]. Utilizing the actuation of mechanical resonance with the same phenomenon demonstrated in [42], Mahboob and Yamaguchi [43] designed a device excited by the piezoelectric effect by applying an AC voltage. In the clamped beam, the application of a DC voltage leads to strain along the beam via the piezoelectric effect enabling the resonant frequency to be tuned. The resonant frequency shift by controlling of the effective spring constant permits the implementation of parametric resonance as well as on-chip electromechanical charge sensing [43]. Piezoelectricity encountered in some materials is one of the unexplored phenomena with potential in nano-scale [264].

### 6.3. Dielectric Tuning Mechanism

Actuation techniques based on dielectric gradient forces, which are becoming more and more powerful tool to tune and control MEMS/NEMS devices [34,332,333]. If the dielectric beam is placed in between two vertically offset electrodes, its vibration can lead to the periodic modulation of their mutual capacitance [334]. Efficient integrated actuation and read-out schemes have been developed to detect the motion of resonators [264]. For example, the resonant frequency can be tuned by capacitive coupling of the nanomechanical element to a side electrode [15]. However, the required metalization of the resonant structure reduces the quality factor significantly via Ohmic losses [335]. For this Faust *et al*. [334] developed an efficient, room-temperature microwave mixing scheme for readout as well as a dielectric drive mechanism to actuate mechanics regardless of the material makeup [34].

**Figure 28:**
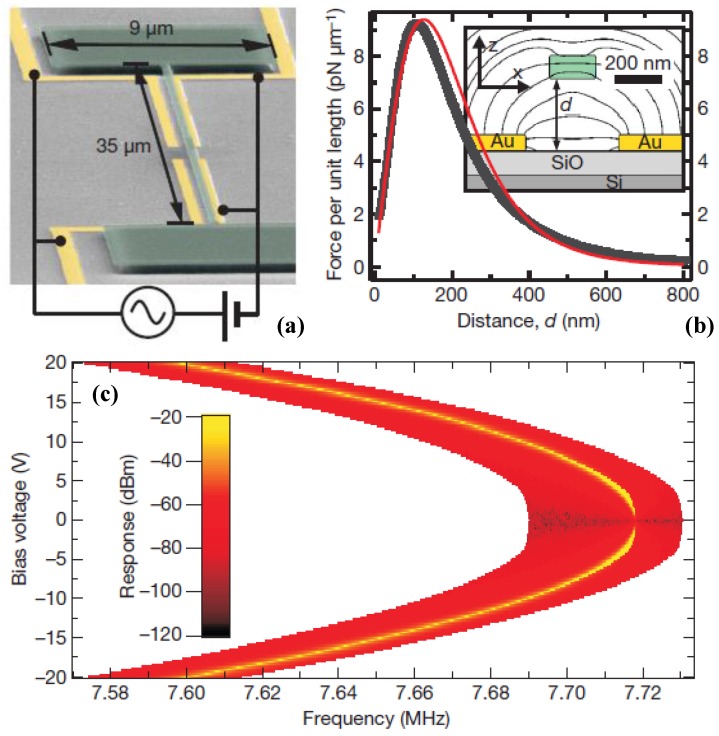
(**a**) SEM of a nanomechanical resonator reported by Unterreithmeier *et al*. [34]; (**b**) The dielectric force acting on the resonator; (**c**) Frequency tuning of the resonator. Reused with permission from [34], Copyright 2009, Nature Publishing Group.

Any polarizable body placed in an inhomogeneous electric field implements a dielectric force, Unterreithmeier *et al*. [34] demonstrated the design of a set of on-chip electrodes to create an electric field gradient and polarize a dielectric resonator and subject it to an attractive force than can be modulated at high frequencies. The mechanism relies mainly on dielectric interaction, in which the polarizable element is a clamped-clamped silicon nitride beam (Figure 28a). The scheme enables simple voltage tuning of the mechanical resonance over a wide frequency range due the dielectric force depending strongly on the resonator-electrode separation. The dielectric force exhibits a maximum at a distance that is comparable, as shown in Figure 28b. The modulation of the resonant frequency can be used to demonstrate parametric actuation [43]. Unterreithmeier *et al*. [34] and Rieger *et al*. [333] found that the force gradient to be proportional to the square of the voltage.

A quadratic dependence of the resonator resonance frequency on the applied dc voltage can be derived from the energy of the induced dipolar moment of the dielectric resonator in an external electric field [333]. The resulting shift in resonant frequency can be expressed as [34]:

(78) $$\Delta f=\frac{1}{2\pi }\sqrt{\frac{k_{0}-c_{u}V_{DC}^{2}}{m}}\approx f_{0}\left(1-\frac{c_{u}V_{DC}^{2}}{2k_{0}}\right)$$

where $c_{u}$ is a constant and relative to the field gradient. The resonance frequency decreases quadratically with bias voltage, as illustrated in Figure 28c. The resonant frequency lies between 5 and 9 MHz and the frequency tuning range of more than 100 kHz, while the quality factor ranges from 100,000 to 150,000 [34]. Rieger *et al*. [333] presented an integrated scheme for dielectric drive and read-out of high-Q nanomechanical resonators that enable tuning of both the resonance frequency and quality factor with an applied DC voltage. The resonance frequencies lie around 6.5 MHz and can be tuned over 5% [333], and the highest quality factor is 340 000 for the out-of-plane mode in the elevated design. Unterreithmeier *et al*. [336] successfully modeled the damping of nanoresonators by postulating a frequency-independent mechanism caused by local strain variation. The large frequency tuning range can be used for *in-situ* tuning of several mechanical elements into resonance [337] or coupling to external elements [338]. In addition, altering the DC voltage does not only shift the resonant frequency, but also influences the dielectrically induced damping which varies quadratically with increasing voltage [15,333].

### 6.4. Magnetomotive Tuning Mechanism

The magnetomotive transduction technique [41,123,339] has played a very important role in the micro- and nanomechanical resonators [1,111], and allows for all-electrical actuation and sensing of the mechanical motion. Although magnetomotive actuation technique is broadband, even in the presence of parasitic capacitances [123], it requires strong magnetic fields, which are usually generated by using superconducting coils [1], and the eddy current damping force caused by this transduction scheme should be examined [10].

**Figure 29:**
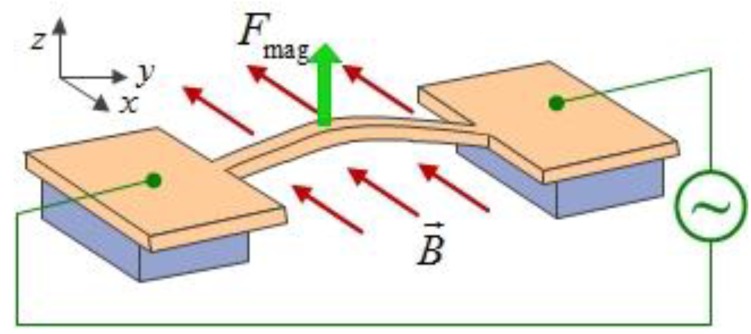
Schematic of magnetomotive actuation for the doubly clamped beam resonator.

The magnetomotive coupling scheme is based on the electrodynamic forces that act on moving charges in a magnetic field [107], and it can be used for SiC and AlN resonator structures. The scheme is shown in Figure 29 in a doubly clamped beam. An external voltage is applied between both contact areas on either side of the freestanding structure. Due to the applied magnetic field B, an alternating Lorentz force is generated in the out-of-plane direction. The Lorentz force $F_{mag}$ depending on the alternating current I can be expressed as:

(79) $$F_{mag}=IBL_{m}$$

where $L_{m}$ is the length of the beam. The induced electromotive force developed along the beam owes to its resulting motion through the magnetic field [41]. A combination of capacitive and magnetomotive excitation for the tunable coupled nanomechanical resonators was reported in [340].

### 6.5. Mode Coupling Tuning Mechanism

As an effective tuning mechanism, the mode coupling of nanomechanical resonators has been intensively investigated during the past several years. Mode coupling can be generally divided into intermodal coupling and the mode coupling between mechanical modes and the other types of modes. The former category mainly includes tension-induced modal coupling [341,342,343,344], the coupling in the single-electron tunneling regime [47,289,293], the coupling in quantum regime [345]. Parametric mode fixing [346] and the mode coupling between different resonators [347,348] belong to the latter. The mode-coupling also provides a dissipation channel for the fundamental mode dynamics in resonators [349]. In addition, according to the coupling strength, mode coupling can be classified into linear coupling [341,342,350] and nonlinear coupling [46,343,344].

In order to clearly understand the coupling between the flexural modes of the resonators, several analytical models were introduced [46,47]. The equation for describing the motion of mode i with considering the modal interactions can be expressed as [47]:

(80) $${\ddot{u}}_{i}+\eta_{i}{\dot{u}}_{i}+\omega_{i}^{2}u_{i}+\sum_{j,k,l}\alpha_{i,j,k,l}u_{j}u_{k}u_{l}=f_{i}\cos(\Omega_{i}t)$$

where $\eta_{i}$, $\omega_{i}$ and $f_{i}$ are the damping, resonant frequency and driving force of mode i, respectively, and the indices j,k,l represents the number of modes considered. It can be simplified to be the Duffing equation of a nonlinear resonator for a single mode at i=j=k=l=1. The parameter α is strongly influenced by the single-electron tunneling processes in the suspended carbon nanotube [293], and the displacement-induced tension in the micromechanical resonators [46].

**Figure 30:**
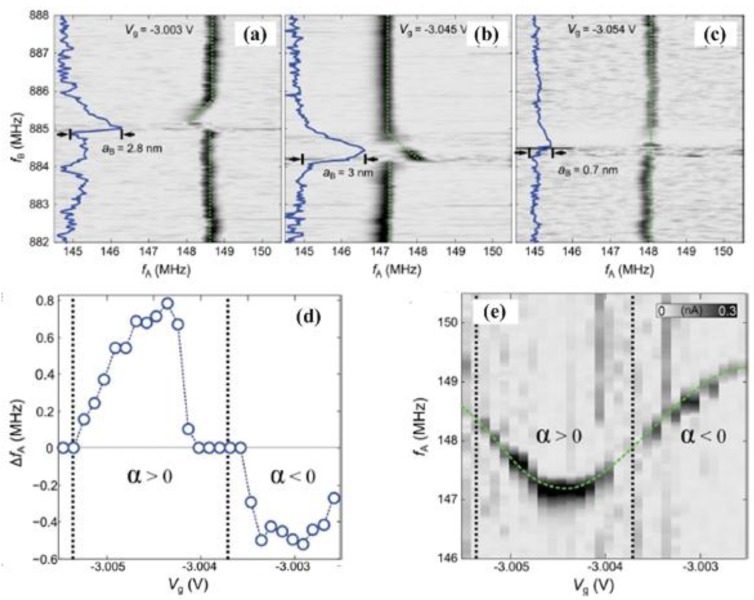
Resonant frequency tuning using the modal interactions reported by Castellanos-Gomez *et al*. [47]. (**a**) Mode softening; (**b**) mode stiffening; (**c**) modal interaction suppression; (**d**) and (**e**) Frequency tuning by the modal interactions as a function of the gate voltage. Reused with permission from [47], Copyright 2012, American Physical Society.

Castellanos-Gomez *et al*. [47] reported that the resonant frequency can be tuned by the modal interactions between the two vibration modes of the carbon nanotube. As shown in Figure 30a–c, three different situations were presented the mechanism, (a) mode softening: the modal interaction reduces the resonant frequency of another mode; (b) mode stiffening: the modal interaction increases the resonance frequency of another mode; and (c) modal interaction suppression: the effect of the modal interaction can be negligible. The modal interaction dominated by single-electron-tunneling processes [293] was verified qualitatively when adjusting the gate voltage, and the frequency tuning by the modal interactions as a function of the gate voltage can be seen in Figure 30d,e. The maximum change in the resonant frequency due to the modal interaction illustrates that a continuous transition from stiffening to softening effects, and the sign of the modal interaction is directly related to that of the nonlinear spring of the carbon nanotube. In addition, the modal interaction strength can continuously by tuned from $\begin{array}{cc} 63\pm 8 & {\text{kHz/nm}}^{\text{2}} \end{array}$ (stiffening) to $\begin{array}{cc} -55\pm 4 & {\text{kHz/nm}}^{\text{2}} \end{array}$ (softening), which is about 6 orders of magnitude larger than that in micromechanical resonators [46]. The nonlinear intrinsic coupling between the flexural–flexural, torsional–torsional and flexural–torsional modes of a microcantilever was experimentally reported in [351]. The direct bending-induced nonlinearities can be identified to facilitate the precise tuning of nanomechanical resonators [352].

### 6.6. Tension-Based Tuning Mechanism

#### 6.6.1. Altering the Effective Length

Frequency tuning of nanomechanical resonators have been previously implemented by altering the effective length of the resonators [353,354,355,356]. Length change is an irreversible process for cantilever beam resonators and is possible only with special geometries for doubly clamped resonators. Jensen *et al* [356] developed a tunable resonator by effectively changing the length of the multiwalled carbon nanotube (MWNT) to tune its resonant frequency. The tunable range is more than 100 MHz. Tension in these devices is applied by the van der Waals attraction between the core nanotube and its shell, and the resonant frequency is approximately calculated by $f_{0}\approx 22.4/(2\pi L^{2})\sqrt{(EI+0.024TL^{2})/\rho A}$. Jensen *et al*. [353] also introduced a nanotube radio that consisted of an antenna and tuner by using a single walled CNT. However, nanoelectromechanical resonators based on these methods have also significant drawbacks because the nanotube was continuously shortened by the trimming process [97,357]. Although the model CNT tuner is difficult to implement by simple processes, it has the advantage that the frequency tuning can be easily obtained via controlling applied voltage without complex nano-positioning platform [357].

#### 6.6.2. Tensile Stress Effect

The resonance frequency is dominated by the large tensile stress [200,358]. By varying the beam stress [359], the resonant frequency of the nanoresonator can be tuned with coving a frequency range from 7 to 206 MHz. The use of axial pre-stress method may allow tuning of the torsional stiffness and resonant frequency of CNTs [360]. The torsional stiffness can increase to approximately 23%. The fact is that the torsional stiffness can be tuning by the axial pre-stress, which indicates nonlinear effects due to mechanical coupling between torsional shear stress and axial pre-stress [361]. However, tensioning methods typically need high DC bias voltages, and the range of frequency tuning is inherently by the bearable tension of the resonant systems [97].

**Figure 31:**
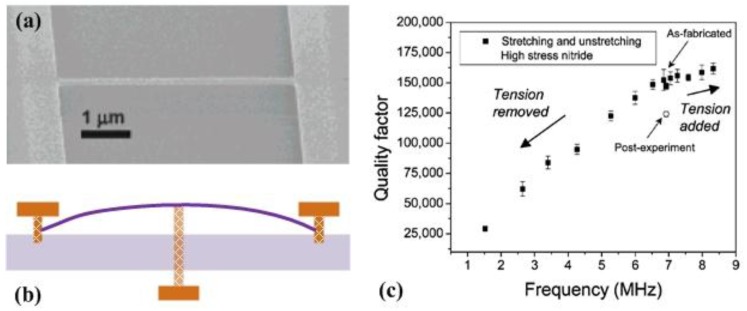
Stress tuning of resonant frequency of the doubly clamped beam resonator reported by Verbridge *et al*. [125]. (**a**) Doubly clamped beam resonator; (**b**) Schematic digram of the added tensile stress to the resonator; (**c**) Experimental results of added stress to tune both resonant frequency and quality factor. Reused with permission from [125], Copyright 2007, American Chemical Society.

Verbridge *et al*. [125,200] demonstrated that the tensile stress can be used as a parameter for achieving increased resonant frequency as well as increased quality factor. The direct stretching technique [125] was developed to provide the ability to dramatically tune both frequency and quality factor of nanomechanical resonators. Figure 31 illustrates the effects of stretching on silicon nitride resonators with two different inherent stress values, and the resonant frequency and quality factor can be controlled over a wide range. For the case of 5 μm long device, the resonant frequency and quality factor change from initial values of 14.6 MHz and 1200 to 35.5 MHz and 6700 at the highest stress value, respectively. The tension of this resonator can also be tuned in both directions. It can enable future mechanical resonators to be used as variable frequency references as well as variable band-pass filters. In addition, the composite buckled beam resonator was designed to provide a very high sensitivity platform for sensing [362,363] and the compressive pre-stress buckles the beam resonator leading to a strong amplitude–frequency relationship [364].

For the case of various boundary conditions, the concept of a local drive force and constraint can allow the cantilever resonator to be tuned over a 300% frequency range [365], but this technique would be difficult to achieve higher-frequency tuability. The shear-strain-induced tension can also become an alternative method for tuning the CNT resonators [366,367].

### 6.7. Active Electrical Tuning Mechanism

Electrical tuning alters the resonant frequency by adjusting the electrical load [60,368]. Circuit-level techniques such as enhanced series tuning have been successfully applied to thin-film piezoelectric-on-silicon resonators [369] and obtaining up to 1500 ppm of tuning [370]. For effective series tuning the resonant frequency of resonators, the parasitic pad capacitances of the resonators were counteracted with negative capacitors [371]. Although electrical tuning is easy to implement and suitable for *in situ* tuning, it has low tuning efficiency.

**Figure 32:**
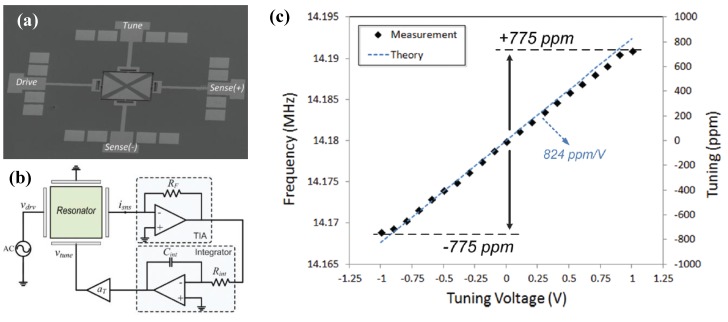
An active feedback-based frequency tuning technique for MEMS resonators reported by Norouzpour-Shirazi *et al.* [371]. (**a**) SEM of the 4-port AlN-on-Si square resonator; (**b**) a feedback loop is used to tune the frequency of the resonator; (**c**) the measured tuning and theoretical values using the active tuning method. Reused with permission from [371], Copyright 2015, IEEE.

More recently, Norouzpour-Shirazi *et al*. [371] reported an active electrical tuning technique for dynamic tuning of MEMS resonators. The basic tuning mechanism is to generate an electrical displacement signal from the resonator output current, and modify the spring constant linearly in either positive or negative directions. This tuning method has been applied to a piezoelectric AIN-on-Si square resonator (Figure 32a). As shown in Figure 32, a tuning feedback loop was used to tune the open-loop resonator frequency response. The resonator was tuned in both positive and negative directions with a linear tuning slope of 830 ppm/V with an overall tuning range of 22 kHz, equivalent to 1550 ppm was measured from active tuning of the closed-loop oscillator.

## 7. Passive Frequency Tuning Methods

In contrast to active frequency tuning methods, passive ones are not able to perform real time frequency tuning throughout the life time of the resonators. However, passive methods require no further energy once the frequency tuning has been employed. Zero on-chip energy consumption makes passive tuning methods attractive for low power applications, such as mechanical filters and oscillators [36]. During the past two decades, some researchers have demonstrated passive methods using tools including selective polysilicon deposition (SPD) [315], focused ion beam (FIB) sputtering or platinum (Pt) deposition [372], electron beam irradiation [373]. Joachim and Lin [315] applied the selective polysilicon deposition (SPD) process to the frequency tuning of comb-drive resonators. The resonant frequency shifts about 1.96% at around 86.6 kHz. The mechanism for changing resonant frequency by the SPD process includes increasing mass and stiffness and altering residual stress. Jaroenapibal *et al*. [373] reported the irreversible frequency tuning scheme of single-walled carbon nanotube (SWNT) bundle resonators via cross-link formations upon electron beam irradiation.

### 7.1. Post-Fabrication Tuning Process

Post-fabrication tuning is implemented to compensate or control for local process variations, as well as defects and errors occurring during manufacturing, which leads to resonant frequency shift. The tuning scheme can also compensate for environmental factors, including ageing [374], contamination [375], and thermal mismatch [298].

**Figure 33:**
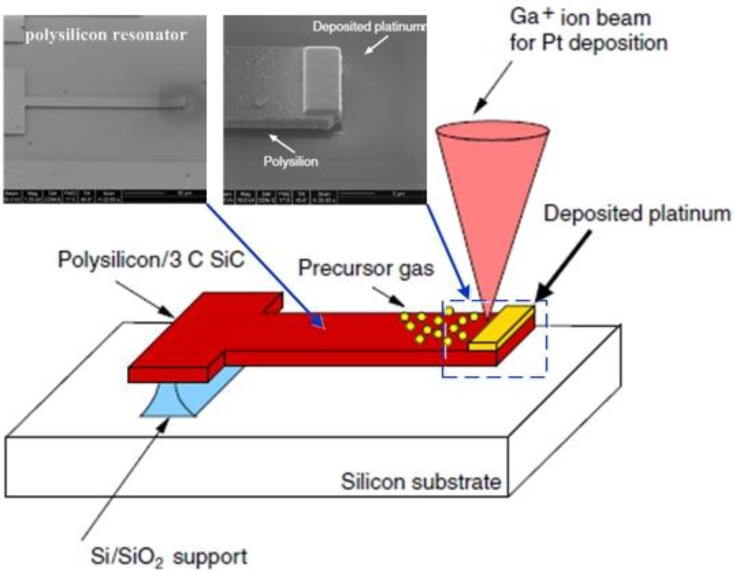
Frequency tuning of resonators by FIB Pt deposition reported by Enderling *et al*. [36]. Reused with permission from IOP Publishing.

Among various passive methods, focused ion beam (FIB) technique has become a powerful tool for the post-fabrication tuning process. FIB can sputter and deposit material in micro- and nano-scales and perform tuning over a wide frequency range without using on-chip power or causing device failure [36,315,372]. Syms and Moore [372] demonstrated an iterative frequency tuning scheme by alternating the stiffness of laterally electrostatic comb-drive microresonators using FIB machining. The resonant frequency shifts about 5% at around 650 Hz. Campanella *et al*. [376] presented a FIB-assisted procedure with the aim of frequency tuning thin-film bulk acoustic resonators. Moore *et al*. [377] monolithically fabricated the MEMS cantilever array in a split ring resonator (SRR) to enable electrostatic tuning of the resonant frequency. The SRR with the FIB cuts on the outer ring has a frequency shift from 14 GHz to around 12.5 GHz. Vick *et al*. [378] presented the introduction of notching into a bulk FIB device strongly tuning the predicted resonant frequency. Adding the notches can reduce the effective stiffness of the resonator and result in reduction of the resonant frequency. Enderling *et al*. [36] reported a novel post-fabrication frequency tuning method for micromechanical cantilever resonators by platinum (Pt) deposition, as illustrated in Figure 33. The FIB Pt deposition on flexural vibrating cantilever beam resonators causes a change in equivalent mass, and can be modeled as spring-mass systems. The change in resonant frequency of cantilevers can be calculated by considering the change in equivalent mass, and the tuning frequency $f_{Pt}$ can be written as [36]:

(81) $$f_{Pt}=\frac{1}{2\pi }\sqrt{\frac{k_{eff}}{m_{eff}+m_{Pt}}}$$

where $m_{Pt}$ is the added Pt mass to the resonator. It can be found that Pt deposits located at the free end of the resonator increase the equivalent mass and decrease the resonant frequency. Although FIB deposition enables the resonant frequency to increase and decrease, Pt mass deposition is often required for resonators with high tensile residual stress [211], which can lead to large increases in resonant frequency and need to be reduced for communication applications [36]. In addition, it requires unceasing power supply to maintain the frequency tuning status [379].

**Figure 34:**
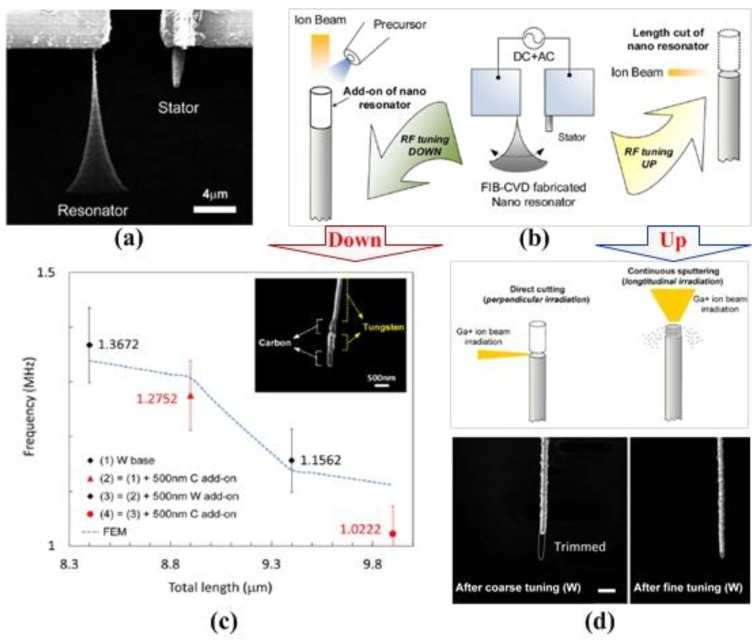
Synthesis and bidirectional frequency tuning of cantilever-shape nanoresonators using FIB-CVD reported by Chang *et al*. [379]. (**a**) Carbon- and tungsten-based nanoresonators under resonance and a stationary stator; (**b**) Scheme of the synthesis and bidirectional frequency tuning mechanism; (**c**) Resonant frequency adjusted by adding material; (**d**) Resonant frequency adjusted by removing materials. Reused with permission from American Chemical Society, Copyright 2013.

Focused-ion-beam chemical-vapor-deposition (FIB-CVD) has advantages and potential in the fabrication of three-dimensional (3D) nanostructures [380]. Chang *et al*. [379] recently demonstrated the synthesis and bidirectional frequency tuning of cantilever-based nanoresonators using FIB-CVD. Single and multi-step frequency tuning processes and schemes were successfully developed for both directions of increasing and decreasing. Figure 34b illustrates the overall scheme of the synthesis and bidirectional frequency tuning of carbon- and tungsten-based nanoresonators under resonance and a stationary stator (Figure 34a). The resonant frequency can be passively adjusted either by adding material to extend the total length of the resonator using the FIB deposition process (Figure 34c) or by removing materials to reduce the entire length using the FIB sputtering process (Figure 34d). On one hand, as shown in Figure 34c, three successive material add-on processes of similar lengths of 500 nm-long carbon and tungsten were conducted on a tungsten nanoresonator with length 8.4 μm and diameter 150 nm to further illustrate the capability of the frequency tuning process. On the other hand, the frequency tuning process to increase the resonant frequency was performed in both coarse and fine tunings as recorded in Figure 34d. More recently, Henze *et al*. [381] demonstrated a post-production hydrofluoric acid etching process for fine-tuning microresonators to reach an arbitrary frequency, and a controllable resonant frequency 10 GHz was observed using this effective approach.

**Figure 35:**
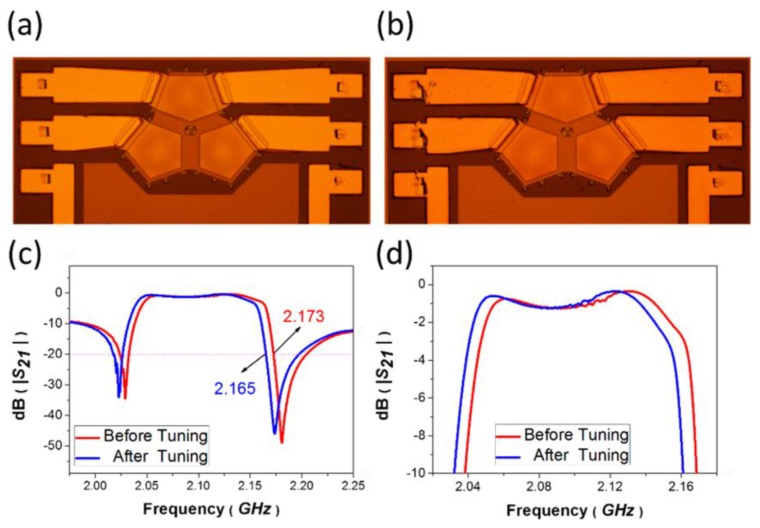
Frequency tuning of microresonator using molecular LbL self-assembly approach reported by Liu *et al*. [382]. Optical microscope images of the FBAR filter (**a**) before and (**b**) after LbL assembly. Electrical performance of (**c**) wide band and (**d**) pass band (red) before and (blue) after LbL assembly. Reused with permission from [382], Copyright 2015, American Chemical Society.

More recently, Liu *et al*. [382] presented a new method to tune the resonant frequency of microfabricated film bulk acoustic resonator (FBAR) using molecular layer-by-layer (LbL) self-assembly approach for wireless broadband communication, as shown in Figure 35. The maximum resonant frequency shift of FBAR reaches more than 20 MHz, meaning 1.4% tunability at least. After 10 bilayers deposition, the pass band shifts down by 8 MHz at −20 dB while the performance in pass band and stop band maintain the same. The frequency tuning method using molecular LbL coating has great advantages because it is a maskless process and the LbL polymer coating offers a direct way to functionalize the resonator surface for receptor based chemical sensing applications.

### 7.2. Post-Packaging Tuning Process

**Figure 36:**
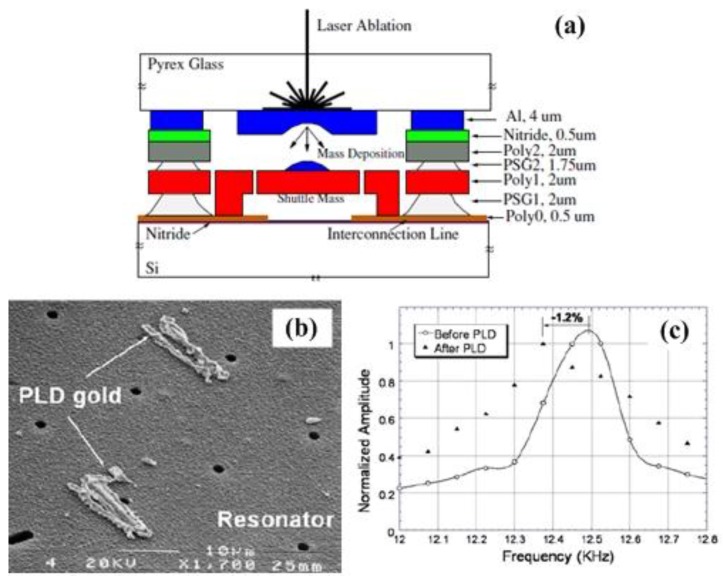
Post-packaging frequency tuning process using PLD [89]. (**a**) Schematic diagram of the PLD tuning process; (**b**) Microresonator surface after PLD tuning process; (**c**) Spectrum measurement of a resonator before and after the PLD frequency tuning process. Reused with permission from IOP Publishing.

Post-fabricating tuning is often needed to obtain uniform properties in the microresonators. Chiao and Lin [89] presented a novel post-packaging tuning of microresonators using pulsed laser deposition (PLD) process, which has the advantages of precise process control, versatility and can be easily implemented into the post-packaging process. The schematic diagram of the post-packaging PLD frequency tuning process is shown in Figure 36a. Since PLD tuning always adds mass to the target surface, the microresonators can be designed to have a higher frequency that can be tuned down to meet the specification. To obtain a well-controlled PLD process, it is necessary to characterize the minimum laser fluence $E_{v}$, which can be expressed as [89]:

(82) $$E_{v}=E_{s}+\frac{H_{l}h}{1-R_{r}}+\frac{c_{pl}(T_{v}-T_{m})h}{1-R_{r}}$$

where $E_{s}$ is the laser fluence, $H_{l}$ is the latent heat of the film, $c_{pl}$ is the specific heat of the molten film, $R_{r}$ is the reflectivity of the metal film, $T_{v}$ and $T_{m}$ are the film boiling and melting temperatures, respectively.

Figure 36b illustrates the microresonator surface after post-packaging PLD tuning process. The PLD gold beads can be identified on the resonator surface as the result of two laser shots at two different locations. From the measured spectrum shown in Figure 36c, it can be observed that the change in resonant frequency of the resonator between before and after the PLD tuning process. Table 9 summarizes and compares some frequency tuning mechanisms and techniques for nanomechanical resonators reported in the literature.

**Table 9:** Comparison of some frequency tuning mechanisms and techniques for nanomechanical resonators. (Note: negative value denotes tuning downward).

Authors	Type	Tuning Mechanism	Tuning Method	Resonant Frequency	Quality Factor	Tuning Range
Jun *et al.* [268]	bilayer beam resonator	Electrothermal tuning (Joule heating)	Active	10.12 MHz	~2000	10%
Jensen *et al.* [356]	Nanotube resonator	Changing the length	Active	~300 MHz	>1000	>100 MHz
Chen *et al.* [147]	Graphene resonator	Electrostatic tuning	Active	52.19 MHz	~55	~14%
Fardindoost *et al.* [383]	Nanofiber resonator	Electrostatic spring-softening effect	Active	580 kHz	2511	−3 kHz
Kwon *et al.* [384]	Nanowire resonator	electrostatic spring-softening effect	Active	1.564 MHz	N/A	2.23%
Chang *et al.* [379]	Carbon-based nanoresonator	FIB-CVD machining	Passive	2.467 MHz	300	−307.7 kHz
	Tungsten-based nanoresonator			1.352 MHz	100	−111.2 kHz
				853.2 kHz		240.4 kHz
Vick *et al.* [378]	Cantilever nanoresonator	Bulk FIB machining	Passive	8.234 MHz	~1623	−7.5%
Rieger *et al.* [333]	Silicon nitride resonator	Dielectric actuation	Active	6.5 MHz	340,000	~5%
Fung *et al* [302]	Nanowire resonator	Electrostatic tuning	Active	59.0 MHz	2200	5 kHz
Ning. *et al.* [212]	Nanotube resonator	Residual tension effect	Active	9.44 MHz	~64.3	11.6 MHz
Verbridge *et al.* [125]	Nanostring resonator	Tensile stress effect	Active	9.3 MHz	105,000	15%
Stiller *et al.* [300]	CNTs resonator	Electrostatic softening effect	Active	358.5 MHz	11,800	−75%

## 8. Concluding Remarks

Micro- and nanomechanical resonators have emerged as robust and ubiquitous devices and serve as important components in advanced technologies ranging from mass sensing and physical, chemical, and biological detections to promise of new materials and observation of quantum phenomena through the change in resonant frequency [385,386,387,388,389,390,391,392,393,394]. The implementations of tunable resonators with desirable frequencies are widely required and provide more opportunities for both scientific and technological applications.

The resonant frequency of a mechanical resonating structure in general scales as 1/L, where L is the scale parameter of the resonator. At microscale, mechanical resonators have their most prominent applications of accurate detection of masses and forces [395]. When the size of mechanical resonators reduces to the nanoscale, high resonant frequencies can be achieved, but the quality factors are also reduced, which makes the arbitrary reduction of the resonator size challenging. In addition, the size reduction can cause the onset of nonlinearity, decrease the dynamic range and make gigahertz-range transduction more complicated [148]. Since the micro- and nanomechanical resonators are characterized by a large surface-to-volume ratio, it is demonstrated that the surface effect has an important role on the resonance behaviors as well as the sensing and detection mechanisms, which are very active and on-going research fields. For example, the investigation of the diffusion of adsorbed atoms over the mechanical resonator surface becomes an increasing interest topic [396,397]. Rarefied gas flows in the transition regime ubiquitously generated by the nanomechanical resonators causes a significant challenge to the fundamental theoretical analysis and physical explanation of the fluid effect and fluid-structure interactions. The intrinsic stress/strain effect on the micro- and nanomechanical resonators should be considered to determine their mechanical performances and resonant frequencies [398]. The shifts in resonant frequency are not only caused by the vibration behavior, but also affected by a variety of physical and chemical factors such as the effects of temperature change, surrounding fluid, humidity and adsorption. It is expected that theoretical and experimental approaches for tuning the resonant frequencies can provide a useful tool for optimizing design of micro- and nanomechanical resonators.

For micro- and nanomechanical resonators, control of vibrational energy dissipation is highly desirable. Energy dissipation in mechanical resonators via several mechanisms, including air damping [399], clamping loss [400], thermoelastic dissipation (TED) [401,402], phonon-tunneling dissipation [403] and Akhiezer effect [404], has been widely and deeply studied. For instance, the clamping losses are a widely damping mechanism in nanomechanical resonators and limit the performance of these resonating devices, and the air damping in micromechanical resonators remains an active area of research. Energy dissipation not only has an important impact on the dynamic behavior of mechanical resonators, but also affects the performance of resonator-based devices. As more and more micro- and nanomechanical resonators operate in the nonlinear regime, nonlinear dissipation leads to a limit to the efficiency of dissipation evading methods and techniques [62,405]. Recently, strong nonlinear dissipation has been observed in CNT and graphene resonators under tensile strain [155], in which the nonlinear dissipation is responsible for destroying the hysteresis. Further systematic investigations should focus on the origins and mechanisms of the nonlinear dissipation and predicting the dominant contribution to dissipation in these resonating devices. Moreover, the dissipative processes and nonlinear dissipations contributing to the resonator performance become the active topic of research. In particular, better dynamic response can be obtained by high frequency and low energy dissipation in the resonators and there have tradeoffs between the resonant frequency and quality factor (dissipation) [274]. Therefore, clearly understanding the energy dissipation mechanisms, further controlling the dissipation, and thus improving the performances of the mechanical resonators, are substantial for implementing their potential applications.

As the geometric dimensions reduce to the microscale and nanoscale, the nonlinearities in most mechanical resonators obviously decrease their frequency stability. And the short-term stability of the resonator is limited by certain noise processes. Although the current frequency stabilization mechanisms provide new strategies for further optimization of micro- and nanomechanical resonators in the nonlinear regime [406], future investigations should focus on the applications of stabilization methods for high frequency nanomechanical resonators. In addition, nonlinearities such as synchronization and chaotic behavior are very important in micro- and nanomechanical resonators [15,24,58,407]. It is necessary to have a fundamental insight into the nonlinear vibration behavior, which may be due to the surface effect. Detecting and exciting the vibration and extending the travel range of the resonators [174,408] should be also paid more attention. The limit of the dynamic range of the micro- and nanomechanical resonators may come from the thermomechanical fluctuations, quantum noise, adsorption and desorption noises, and extrinsic vibrational and noise sources [15,181,409]. The present linear dynamic range of such resonating devices is useful but severely limited, and even operating in the nonlinear regime. Dynamic control of resonant frequencies and widening the dynamic range of mechanical resonators are the significant and challenging tasks for emerging applications in MEMS/NEMS.

As the first experimental realization of a mechanical resonator in the coupled quantum system was reported by O’Connell *et al*. [410], the mechanical resonators have attracted more and more attentions [64] due to their the advantages such as superposition and coherent control of the quantum states. The occurrence of nonplanar motions in nanotube and nanowire resonators can be used to broaden interesting applications [411]. Graphene mechanical resonators can provide the required frequency tunability. The development of suspended micro- and nanochannel resonators will make the measurement with a resolution on the 10 ag scale possible. More significantly, the self-assembled molecular structure reported in [35] provides a novel way to characterize the mechanical resonators, and will cause challenges to extensively explore a variety of potential applications [412]. Except for the experimental researches, more theoretical explanations and new computational models should be developed to focus on the underlying mechanisms and make clear understanding of the complex coupling effects on the resonance behavior and performance of the mechanical resonators.

Recent advances of tunable resonators make the fundamental understanding of the frequency tuning mechanisms is important for the future design and optimization of VHF and UHF micro- and nanomechanical resonators. The methodologies of resonant frequency tuning for micro- and nanomechanical resonators contain active and passive methods. One one hand, although previous active tuning approaches such as electrothermal, electrostatic, piezoelectrical, dielectric and magnetomotive techniques are able to tune the resonant frequency of the resonators very precisely, they suffer from either higher power consumption and lower power efficiency or poor nanoscale control over electromechanical coupling effect. Alternative actuation schemes may play an important role in future works as well. On the other hand, the passive methods are preferable in low power consumption applications. Previous passive tuning methods have disadvantageous challenges such as needing expensive equipment and time consuming processes. It is expected that the new passive tuning methods not only have enabled to fabricate micro- and nanomechanical resonators, but also have the capability of conducting efficient and precise frequency tuning, and have not limited to one-direction.

In this review, we have presented an overview of theoretical and experimental approaches which have been used to get insights into the underlying frequency tuning mechanisms of micro- and nanomechanical resonators as well as their wide range of applications. We have briefly described the resonance behavior and frequency tuning principle depending on the change either the stiffness or the mass of the mechanical resonators, and reviewed some tuning structures and latest research progress on the mechanical resonators. We have also discussed some major influencing factors on the implementations of resonators. The efforts to predict, control and apply the resonant frequency shift in the micro- and nanomechanical resonators have been demonstrated. A comprehensive review of research progress on the active and passive frequency tuning methods and techniques for the micro- and nanomechanical resonators has been detailedly addressed. We have additionally provided extensive discussion of the challenges and potential research directions to the tunable micro- and nanomechanical resonators. We hope this review would be helpful for better understanding the importance of frequency tuning in developing the next generation of micro- and nanomechanical resonators.

## Abbreviations

BG	bottom-gate	Pt	platinum
CNT	carbon nanotube	RF	radio frequency
EMF	electromotive force	RTA	rapid thermal anneal
EG	end-gate	SPD	selective polysilicon deposition
FBAR	film bulk acoustic resonator	SWNT	single-walled carbon nanotube
FIB	Focused-ion-beam	SRR	split ring resonator
FIB-CVD	FIB chemical- vapor- deposition	SEM	Scanning Electron Microscopy
LbL	layer-by-layer	TEC	thermal expansion coefficients
MEMS	micro-electro-mechanical systems	TED	thermoelastic dissipation
NEMS	nano-electro-mechanical systems	3D	three-dimensional
MWNT	multiwalled carbon nanotube	VHF	very-high frequency
PLD	pulsed laser deposition	UHF	ultra-high frequency

## Nomenclature

$A_{dc}$	static deflection amplitude	$K_{f}$	spring constant
$A_{e}$	elastic hardening tuning coefficient	$K_{C}$	capacitance expansion coefficient
$A_{t}$	cross-sectional area	$k_{1}$	is the linear spring stiffness
B	applied magnetic field	$k_{p}$	spring constant
$B_{c}$	capacitive softening coefficient	$K_{e}$	effective thermal conductivity
$c_{h}$	heat transfer coefficient	$K_{t}$	torsional spring constant
$c_{p}$	weighted average of heat capacity	$L_{b}$	equivalent length of the bar
$c_{pl}$	specific heat of the molten film	$L_{m}$	length of the beam
$c_{t}$	weighted average of thermal conductivity	$m_{\text{eff}}$	effective mass
$c_{u}$	field gradient constant	$m_{f}$	resonator mass
C	capacitance per unit area	$m_{Pt}$	added Pt mass
$C_{f}$	damping	n	electrostatic dimension parameter
$C_{g}$	capacitance	p	nonlinearity of order
$C_{p}$	specific heat conductivity	$P_{e}$	effective electrical heat production
$C^{\prime }$, $C^{\prime \prime }$	first and second derivative of capacitances	$P_{\text{laser}}$	incident optical power
$d_{3j}$	anisotropic piezoelectric coefficient	$P^{\prime }$	Joule heating rate
$d_{t}$	total thickness	r	vector in the plane
$d_{w}$	diameter of the cylindrical wire	$r_{o}$	experimental optical spot size
E	Young’s modulus	$R_{C}$	resistance
$E_{s}$	laser fluence	$R_{r}$	reflectivity
$E_{v}$	minimum laser fluence	$t_{p}$	total device thickness
$f_{0}$	fundamental resonant frequency	$T_{0}$	ambient temperature
$f_{i}$	initial measured frequency	$T_{d}$	effective dielectric thickness
$F_{e}$	external force	$T_{f}$	thermal axial load
$F_{mag}$	Lorentz force	$T_{m}$	film melting temperature
$\bar{f}$	average frequency	$T_{v}$	film boiling temperature
$G_{s}$	modulus of elasticity in shear	$T^{\prime }$	derivative of temperature
H	thickness	V	applied voltage
$H_{l}$	latent heat	$V_{dc}$	dc voltage
I	electrical current	$V_{g}$	applied voltages on the gate
$I_{m}$	maximum intensity	$V_{tun}$	tuning voltage
$I_{p}$	polar moment of inertia of the area	x	displacement
$I_{t}$	inertial of the paddle	y	deflection
$k_{0}$	mechanical stiffness	$y_{0}$	initial air-gap
$k_{\text{eff}}$	effective stiffness	$Z_{dc}$	static displacement
$k_{total}$	total stiffness		

## Greek Symbols

α	thermal expansion coefficient	ρ	density
$\alpha_{e}$	effective thermal expansion coefficient	$\rho_{p}$	weighted average of mass density
$\beta_{e}$	geometrical constant	$\delta_{L}$	change in length
$\gamma_{T}$	net heat loss rate	ϕ	potential
ϑ	experimental power coefficient	ν	Poisson’s ratio
$\kappa_{T}$	thermal conductivity	$\omega_{e}$	frequency
σ	tensile stress	ξ	variable
$\sigma_{i}$	initial tensile stress	Δf	frequency change
$\sigma_{a}$	axial stress	Δk	stiffness offset coefficient
$\sigma_{th}$	temperature-dependent thermal stress	Δm	mass offset coefficient
$\sigma_{T}$	thermal stress	ΔT	uniform change
